# Supplementary material for: Noradrenergic signaling mediates cortical early tagging and storage of remote memory
Source: Nat Commun. 2022 Dec 9;13:7623. doi: 10.1038/s41467-022-35342-x (PMC9734098; doi:10.1038/s41467-022-35342-x)
Supplement: Supplementary file 1 — Supplementary Information [file 41467_2022_35342_MOESM1_ESM.pdf]

Supplementary figures and figure legends

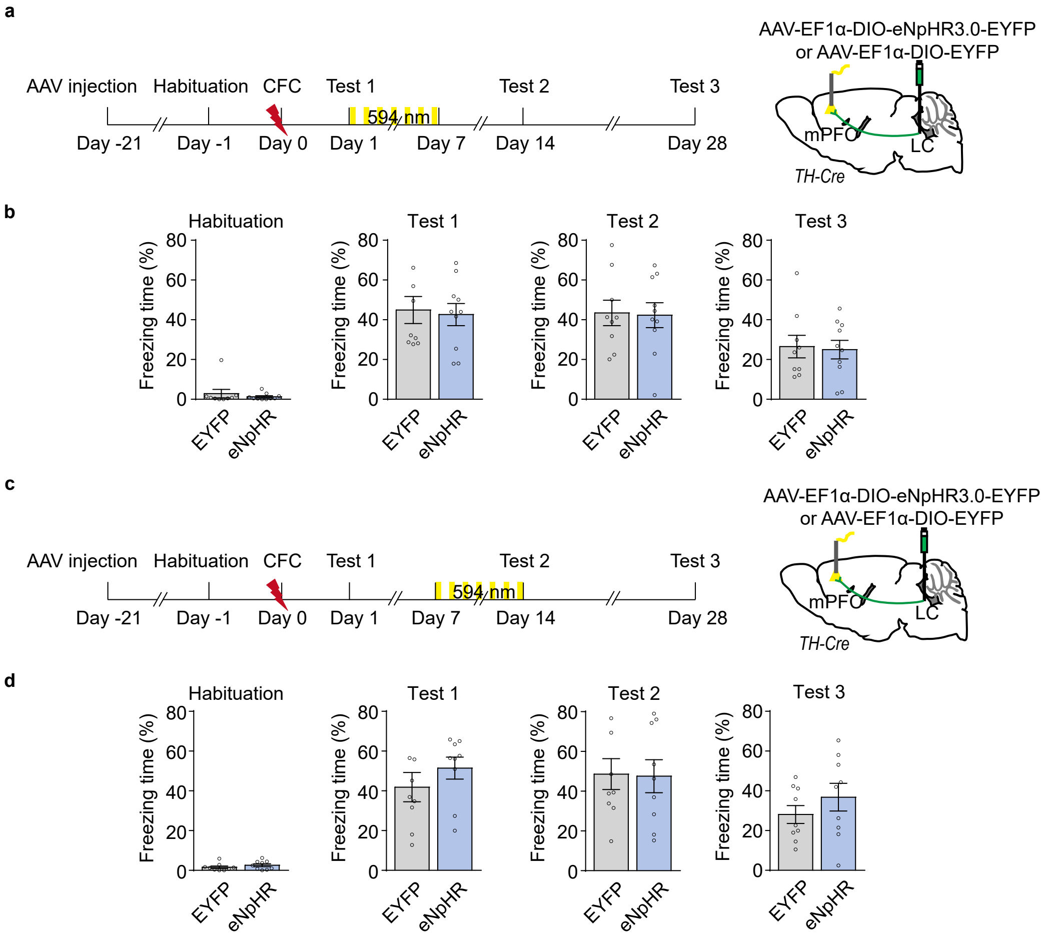

Supplementary Fig. 1 Inhibition of LC-mPFC NEergic projection during post-conditioning period does not impair remote memory storage. a Experimental scheme. *AAV-EF1α-DIO-eNpHR3.0-EYFP* or *AAV-EF1α-DIO-EYFP* was injected in the LC of *TH-Cre* mice, and optical fibers were implanted above the mPFC. 594 nm laser stimulation was daily delivered from 1st day to 7th day after CFC (594 nm, 5 mW, 10 min). **b** Statistical graphs for freezing levels in memory tests [EYFP: $n = 9$; eNpHR: $n = 10$]. **c** Experimental scheme. Same with **a**. Optogenetic stimulation was daily delivered from 8th day to 14th day after CFC (594 nm, 5 mW, 10 min). **d** Statistical graphs for freezing levels in memory tests [EYFP: $n = 9$; eNpHR: $n = 9$].

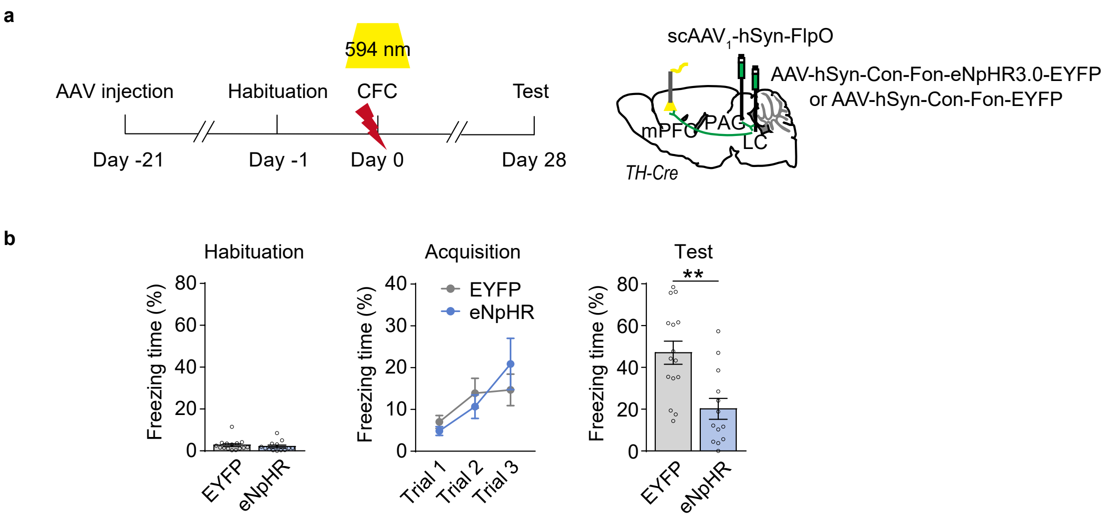

Supplementary Fig. 2 Inhibition of LC-mPFC NE projection innervated by the PAG during CFC training impaired remote memory storage on Day 28. a Experimental scheme. Anterograde *scAAV$_1$-hSyn-FlpO* was injected in the vlPAG, *AAV-hSyn-Con-Fon-eNpHR3.0-EYFP* or *AAV-hSyn-Con-Fon-EYFP* was injected in the LC of *TH-Cre* mice, and optical fibers were implanted above the mPFC. The 594 nm laser was delivered during CFC training. **b** Statistical graphs for freezing levels during memory tests [EYFP: $n = 15$; eNpHR: $n = 13$; Test: $t_{(26)} = 3.57$, $p = 0.0014$]. $^{**}p < 0.01$ vs indicated group.

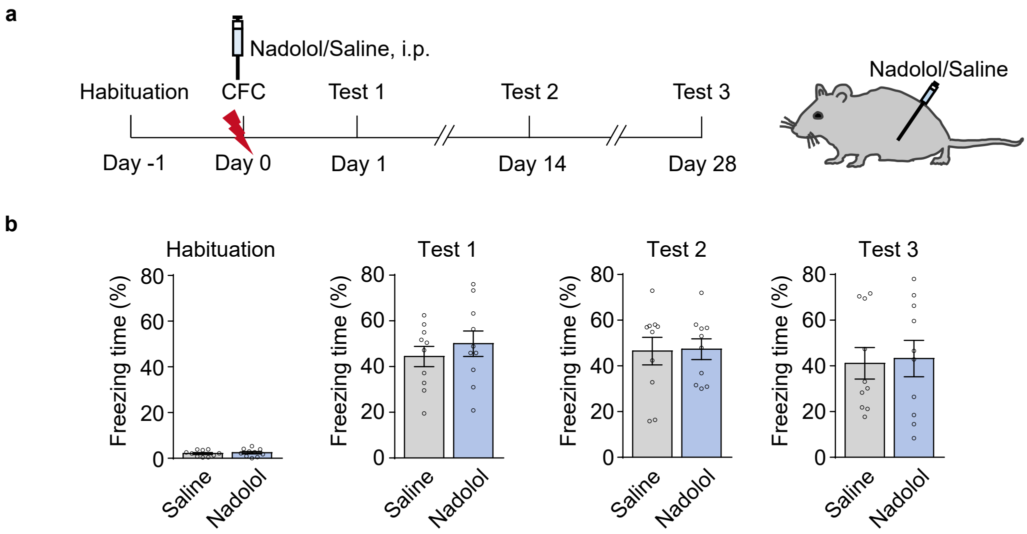

Supplementary Fig. 3 Nadolol does not inhibit remote memory storage. a Experimental scheme. Nadolol (5 mg/kg, i.p.) was injected 30 min before CFC. **b** Statistical graphs for freezing levels during memory tests [Saline: n = 10; Nadolol: n = 10].

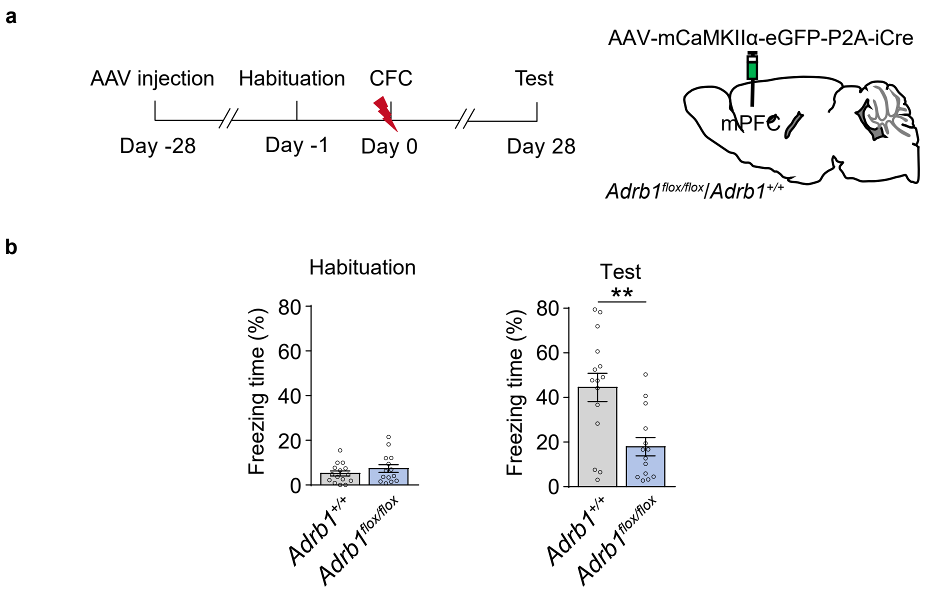

Supplementary Fig. 4 *Adrb1* deletion in mPFC glutamatergic neurons impaired remote memory storage on Day 28. a Experimental scheme. *AAV-mCaMKIIα-eGFP-P2A-iCre* was injected in the mPFC of *Adrb1*$^{flox/flox}$ and *Adrb1*$^{+/+}$ mice. Behavioral tests were performed 1 month after virus injection. Memory retention test was performed on Day 28. **b** Statistical graphs for freezing levels during memory tests [*Adrb1*$^{+/+}$: $n = 15$; *Adrb1*$^{flox/flox}$: $n = 14$; Test: $U = 41$, $p = 0.0043$]. $^{**}p < 0.01$ vs indicated group.

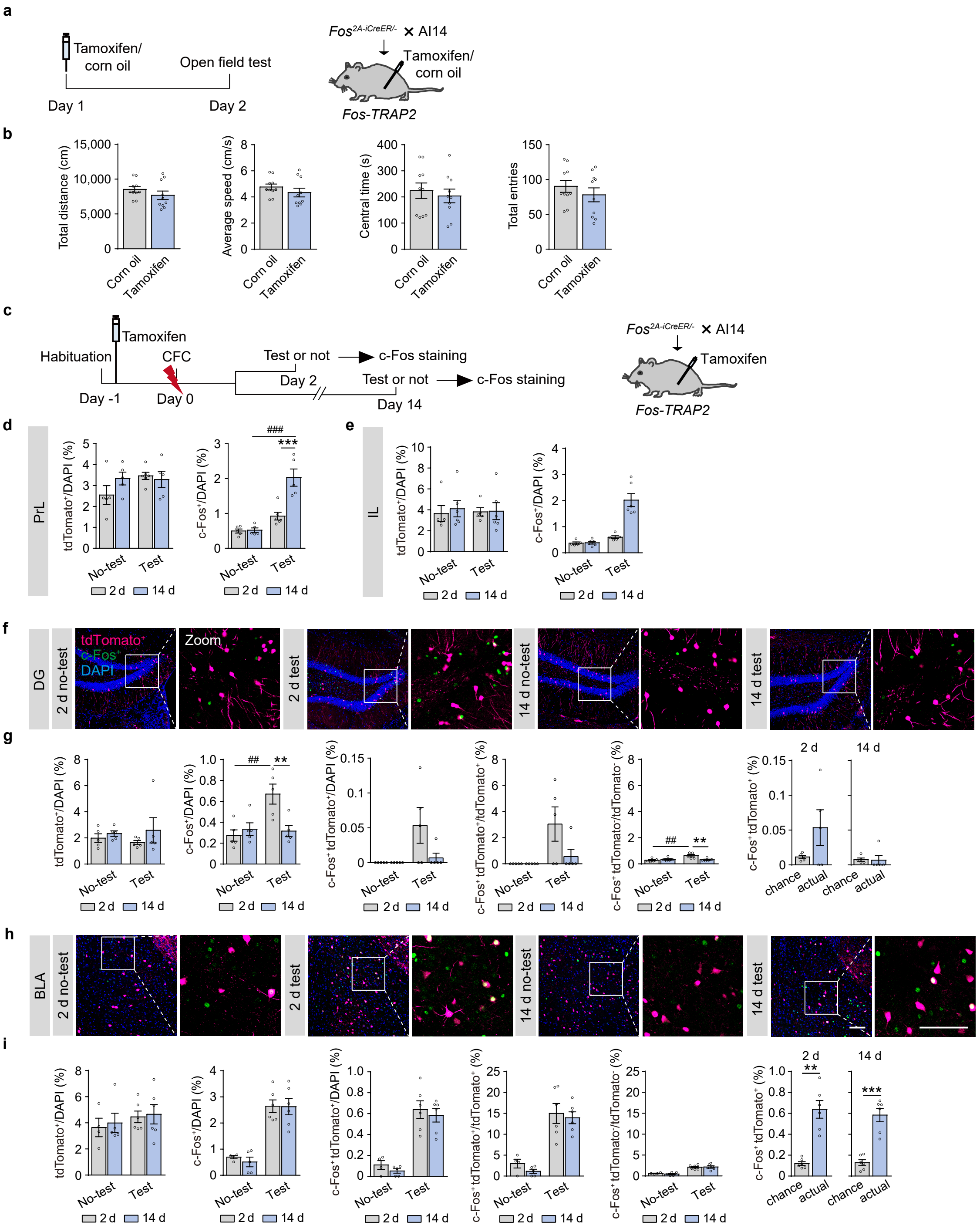

Supplementary Fig. 5 The activity of engram cells after recent and remote memory retrieval in *Fos-TRAP2* mice. a Experimental scheme. In *Fos*$^{2A\text{-}iCreER}$*::AI14* mice, tamoxifen or corn oil was intraperitoneally injected 24 h before Open field tests (Tamoxifen: 125 mg/kg, i.p.). **b** The statistical graphs for total distance and average speed in the box, and time spend and entries to the central zone during open field tests [Corn oil: *n* = 10; Tamoxifen: *n* = 10]. **c** In *Fos*$^{2A\text{-}iCreER}$*::AI14* mice, tamoxifen was

intraperitoneally injected 24 h before CFC (125 mg/kg, i.p.). Memory tests were carried out 2 days or 14 days after CFC, and the mice were perfused 90 min after memory test for c-Fos immunostaining. **d,e** The statistical graphs for $tdTomato^{+}$ and $c\text{-}Fos^{+}$ cell counts in the PrL (**d**) and IL (**e**) [PrL: 2 d no-test: $n = 5$, 14 d no-test: $n = 5$, 2 d test: $n = 6$, 14 d test: $n = 5$; $c\text{-}Fos^{+}$/DAPI: $F_{treatment\times time(1,17)} = 14.92$, $p = 0.0012$. IL: 2 d no-test: $n = 5$, 14 d no-test: $n = 6$, 2 d test: $n = 5$, 14 d test: $n = 6$]. **f,h** Representative images. **g,i** The statistical graphs for $tdTomato^{+}$, $c\text{-}Fos^{+}$, $c\text{-}Fos^{+}$ $tdTomato^{+}$ cell counts and percentage of $c\text{-}Fos^{+}$ $tdTomato^{+}$, $c\text{-}Fos^{+}$ $tdTomato^{-}$ cells in the DG and BLA [DG: 2 d no-test: $n = 5$, 14 d no-test: $n = 5$, 2 d test: $n = 5$, 14 d test: $n = 5$; $c\text{-}Fos^{+}$/DAPI: $F_{treatment\times time(1,16)} = 9.24$, $p = 0.0078$; $c\text{-}Fos^{+}$ $tdTomato^{-}$/$tdTomato^{-}$: $F_{treatment\times time(1,16)} = 7.83$, $p = 0.013$. BLA: 2 d no-test: $n = 4$, 14 d no-test: $n = 5$, 2 d test: $n = 6$, 14 d test: $n = 6$; 2d $c\text{-}Fos^{+}$ $tdTomato^{+}$: $t_{(5.49)} = 6.00$, $p = 0.0013$; 14d $c\text{-}Fos^{+}$ $tdTomato^{+}$: $t_{(10)} = 6.50$, $p < 0.001$]. Scale bar: 100 μm. $^{**}p < 0.01$, $^{\#\#}p < 0.01$, $^{***}p < 0.001$, $^{\#\#\#}p < 0.001$ vs indicated group.

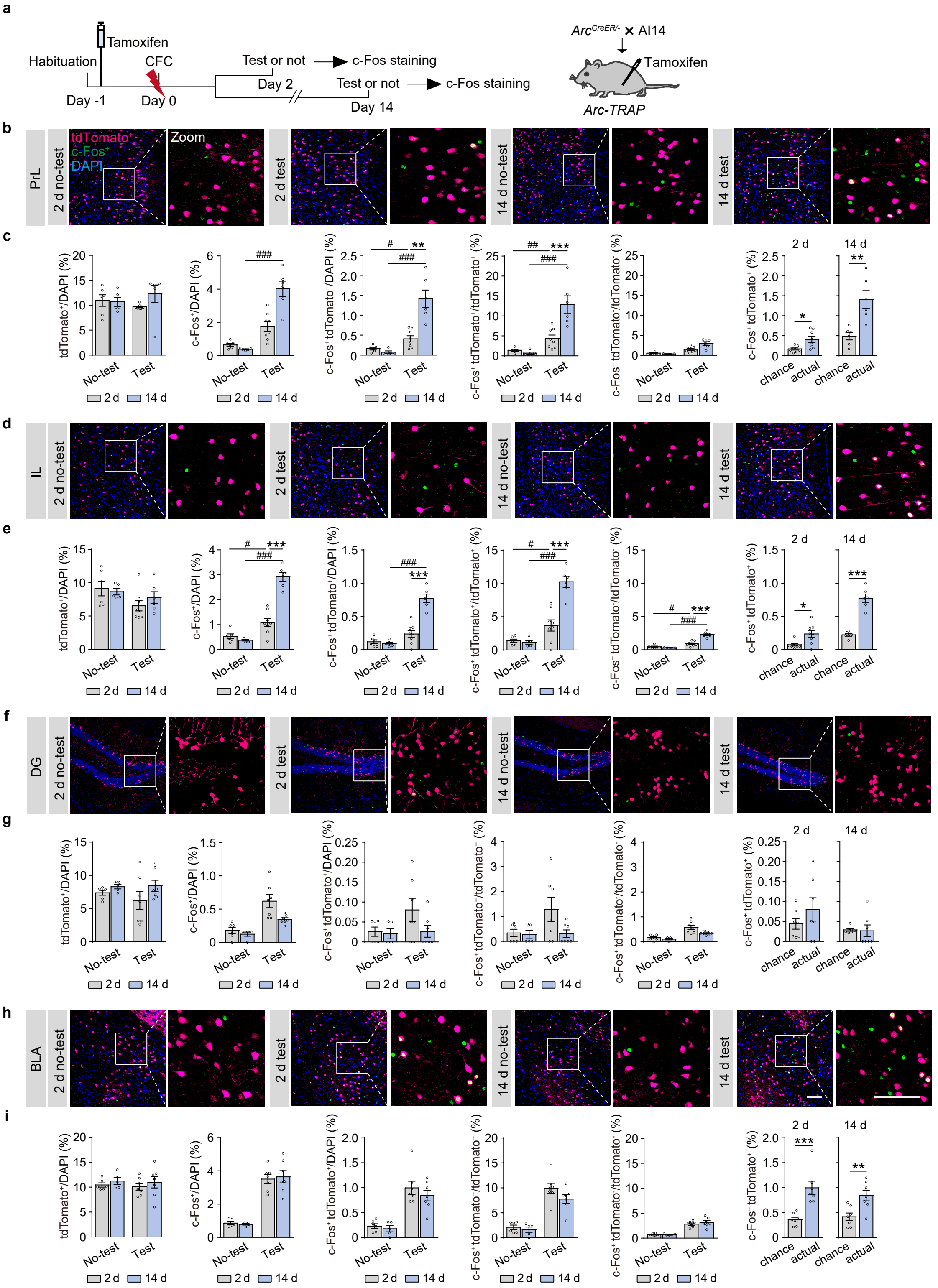

Supplementary Fig. 6 The activity of engram cells after recent and remote memory retrieval in *Arc-TRAP* mice. a Experimental scheme. In Arc^{CreER}*::AI14* mice, tamoxifen was intraperitoneally injected 24 h before CFC (125 mg/kg, i.p.). Memory tests were carried out 2 days or 14 days after CFC, and the mice were perfused 90 min

after memory test for c-Fos immunostaining. **b,d,f,h** Representative images. **c,e,g,i** The statistical graphs for tdTomato$^+$, c-Fos$^+$, c-Fos$^+$ tdTomato$^+$ cell counts and percentage of c-Fos$^+$ tdTomato$^+$, c-Fos$^+$ tdTomato$^-$ cells in the PrL (**c**), IL (**e**), DG (**g**) and BLA (**i**) [PrL: 2 d no-test: $n = 6$, 14 d no-test: $n = 5$, 2 d test: $n = 8$, 14 d test: $n = 6$; c-Fos$^+$/DAPI: $H_{\text{treatment×time}(1,21)} = 4.28$, $p = 0.039$; c-Fos$^+$ tdTomato$^+$/DAPI: $H_{\text{treatment×time}(1,21)} = 4.37$, $p = 0.037$; c-Fos$^+$ tdTomato$^+$/tdTomato$^+$: $H_{\text{treatment×time}(1,21)} = 4.18$, $p = 0.041$; 2d c-Fos$^+$ tdTomato$^+$: $t_{(8.66)} = 2.93$, $p = 0.017$; 14d c-Fos$^+$ tdTomato$^+$: $t_{(10)} = 3.88$, $p = 0.0031$. IL: 2 d no-test: $n = 6$, 14 d no-test: $n = 5$, 2 d test: $n = 8$, 14 d test: $n = 6$; c-Fos$^+$/DAPI: $F_{\text{treatment×time}(1,21)} = 47.11$, $p < 0.001$; c-Fos$^+$ tdTomato$^+$/DAPI: $F_{\text{treatment×time}(1,21)} = 32.31$, $p < 0.001$; c-Fos$^+$ tdTomato$^+$/tdTomato$^+$: $F_{\text{treatment×time}(1,21)} = 22.93$, $p < 0.001$; c-Fos$^+$ tdTomato$^-$/tdTomato$^-$: $F_{\text{treatment×time}(1,21)} = 35.81$, $p < 0.001$; 2d c-Fos$^+$ tdTomato$^+$: $U = 10$, $p = 0.021$; 14d c-Fos$^+$ tdTomato$^+$: $t_{(6.00)} = 8.36$, $p = 0.0002$. DG: 2 d no-test: $n = 6$, 14 d no-test: $n = 5$, 2 d test: $n = 7$, 14 d test: $n = 7$. BLA: 2 d no-test: $n = 6$, 14 d no-test: $n = 5$, 2 d test: $n = 7$, 14 d test: $n = 7$; 2d c-Fos$^+$ tdTomato$^+$: $U = 0$, $p = 0.0006$; 14d c-Fos$^+$ tdTomato$^+$: $t_{(12)} = 3.23$, $p = 0.0073$.]. Scale bar: 100 μm. $^*p < 0.05$, $^\#p < 0.05$, $^{**}p < 0.01$, $^{\#\#}p < 0.01$, $^{***}p < 0.001$, $^{\#\#\#}p < 0.001$ vs indicated group.

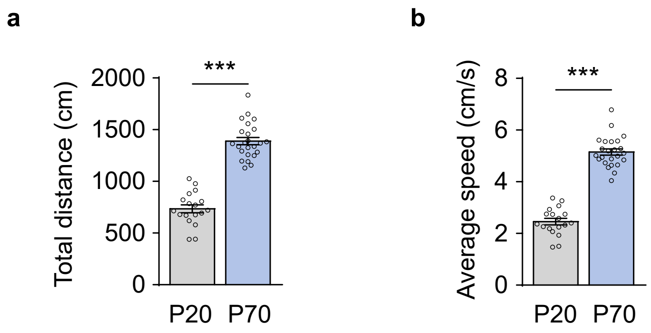

Supplementary Fig. 7 The locomotor activity of juvenile (P20) and adult (P70) mice. a Statistical graph for total distance that mice traveled during habituation [P20: $n = 18$; P70: $n = 24$; $t_{(40)} = 12.54$, $p < 0.001$]. **b** Statistical graph for average speed during habituation [P20: $n = 18$; P70: $n = 24$; $t_{(40)} = 15.17$, $p < 0.001$]. $^{***}p < 0.001$ vs indicated group.

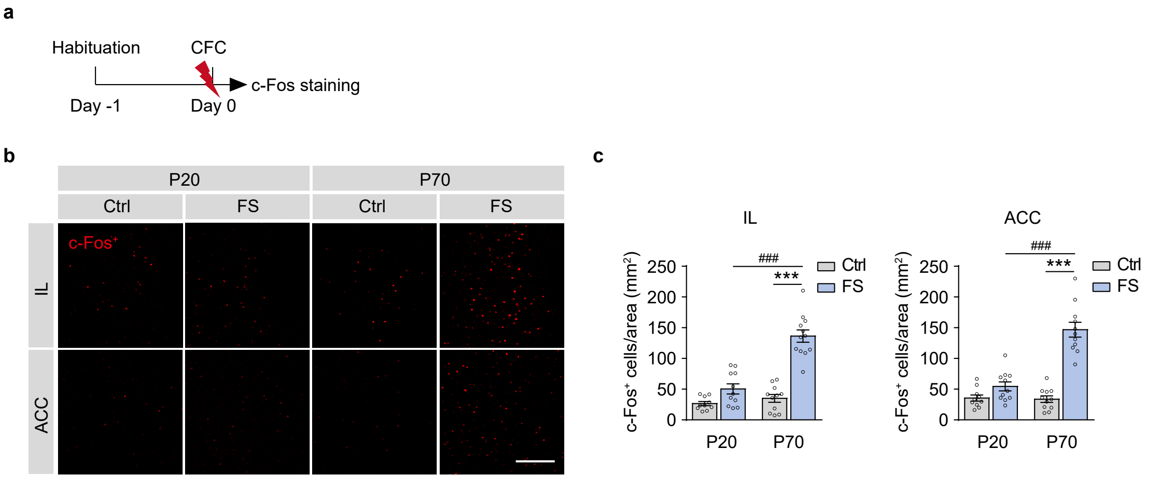

Supplementary Fig. 8 C-Fos expression does not change after CFC in the IL and ACC of juvenile mice. a Experimental scheme. Juvenile and adult mice were perfused 90 min after CFC for c-Fos immunostaining. **b** Representative images. **c** Statistical graphs for c-Fos^{+} cell counts [IL: P20/Ctrl: $n = 10$; P20/FS: $n = 11$; P70/Ctrl: $n = 11$; P70/FS: $n = 12$; $F_{\text{age×treatment}(1,40)} = 25.25$, $p < 0.001$. ACC: P20/Ctrl: $n = 10$; P20/FS: $n = 11$; P70/Ctrl: $n = 11$; P70/FS: $n = 11$; $F_{\text{age×treatment}(1,39)} = 33.92$, $p < 0.001$]. Scale bar: 200 µm. $^{***}p < 0.001$, $^{\#\#\#}p < 0.001$ vs indicated group.

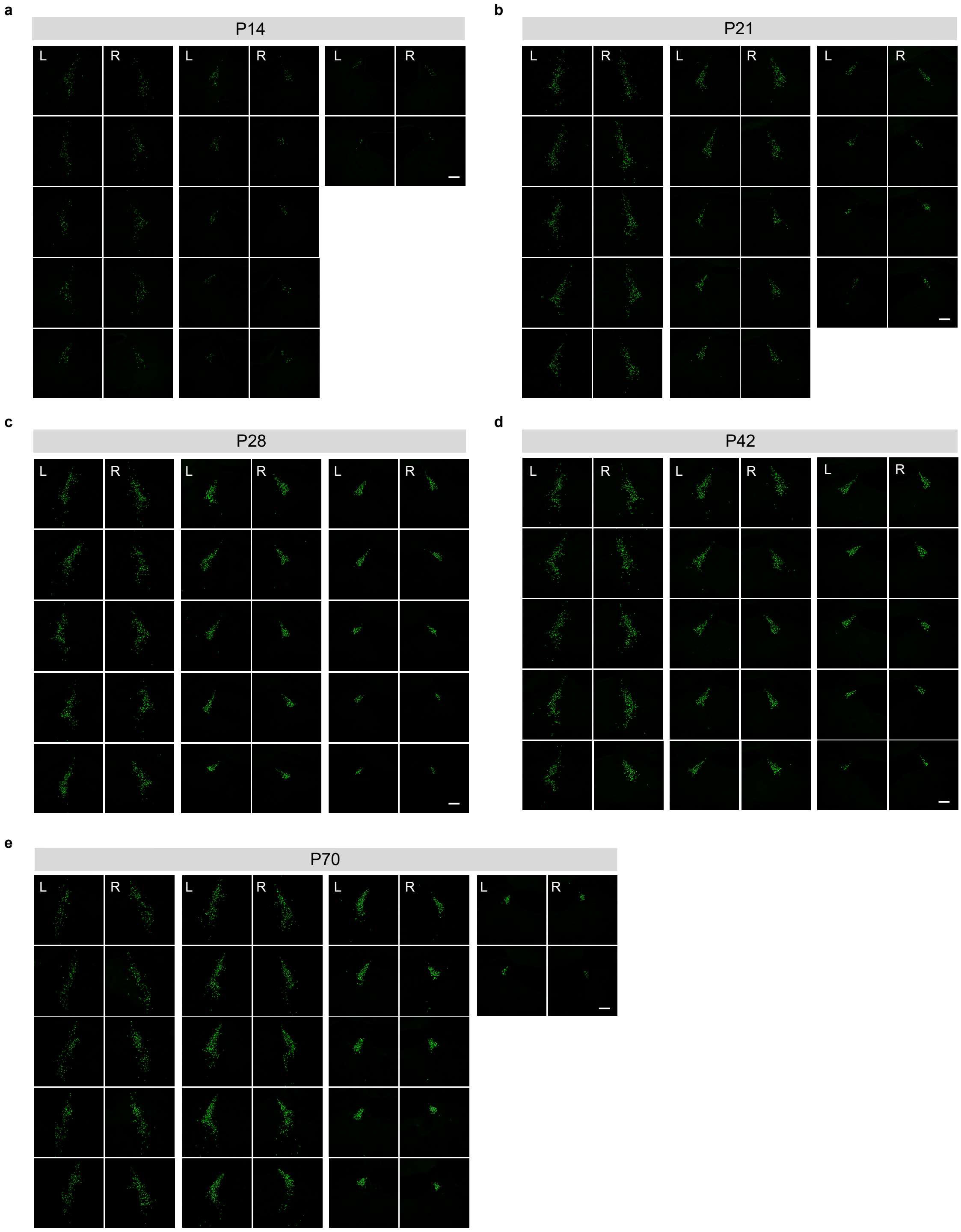

Supplementary Fig. 9 Dbh$^+$ neurons in the LC of juvenile and adult mice. Representative images of H2B-GFP$^+$ cells in the entire LC of *Dbh::H2B-GFP* mice (P14, P21, P28, P42, P70). Scale bar: 200 μm.

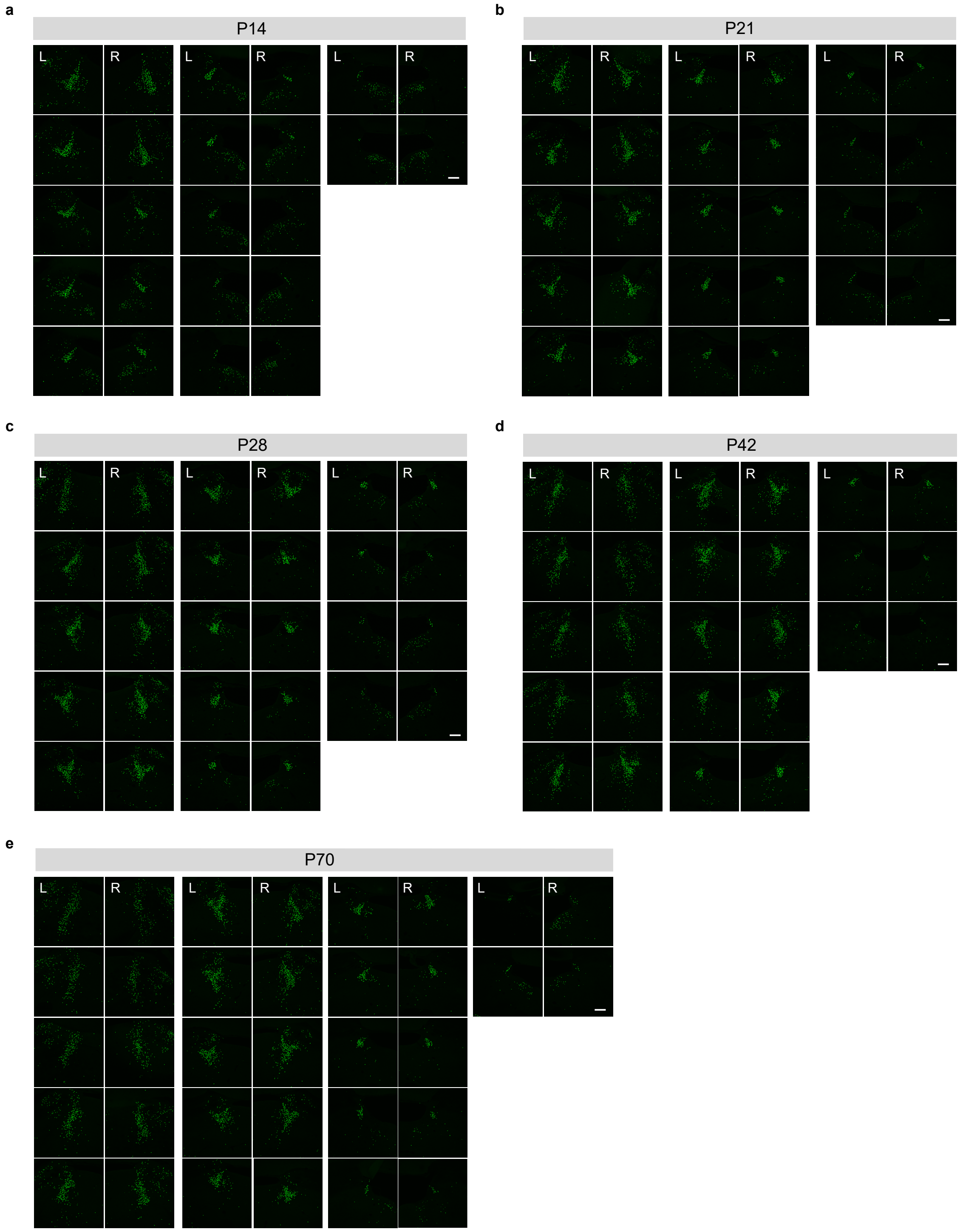

Supplementary Fig. 10 TH^{+} neurons in the LC of juvenile and adult mice. Representative images of H2B-GFP^{+} cells in the entire LC of *TH::H2B-GFP* mice (P14, P21, P28, P42, P70). Scale bar: 200 μm.

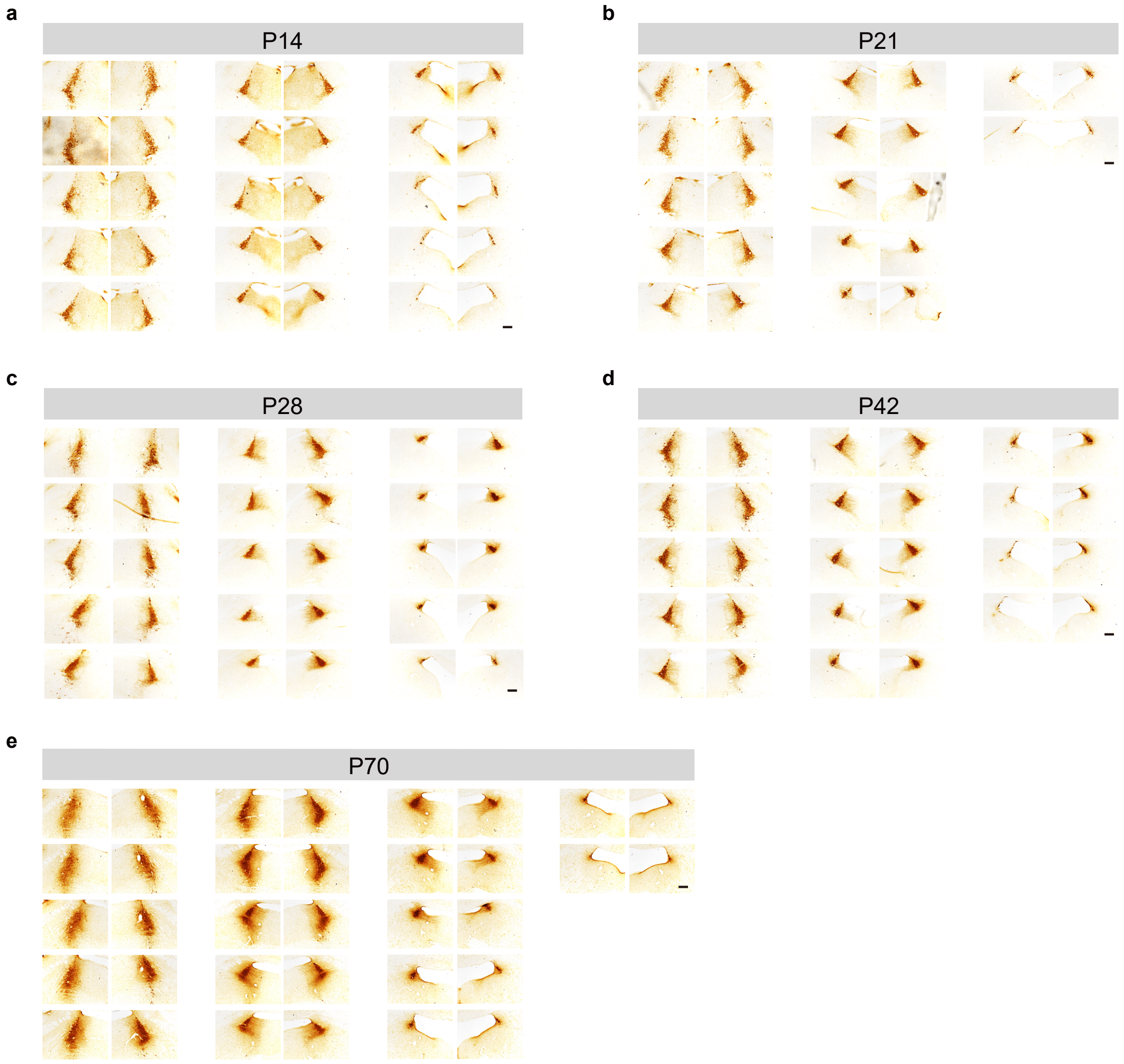

Supplementary Fig. 11 TH volume in the LC of juvenile and adult mice. TH immunostaining was performed in the mice with different ages (P14, P21, P28, P42, P70). Scale bar: 200 μm.

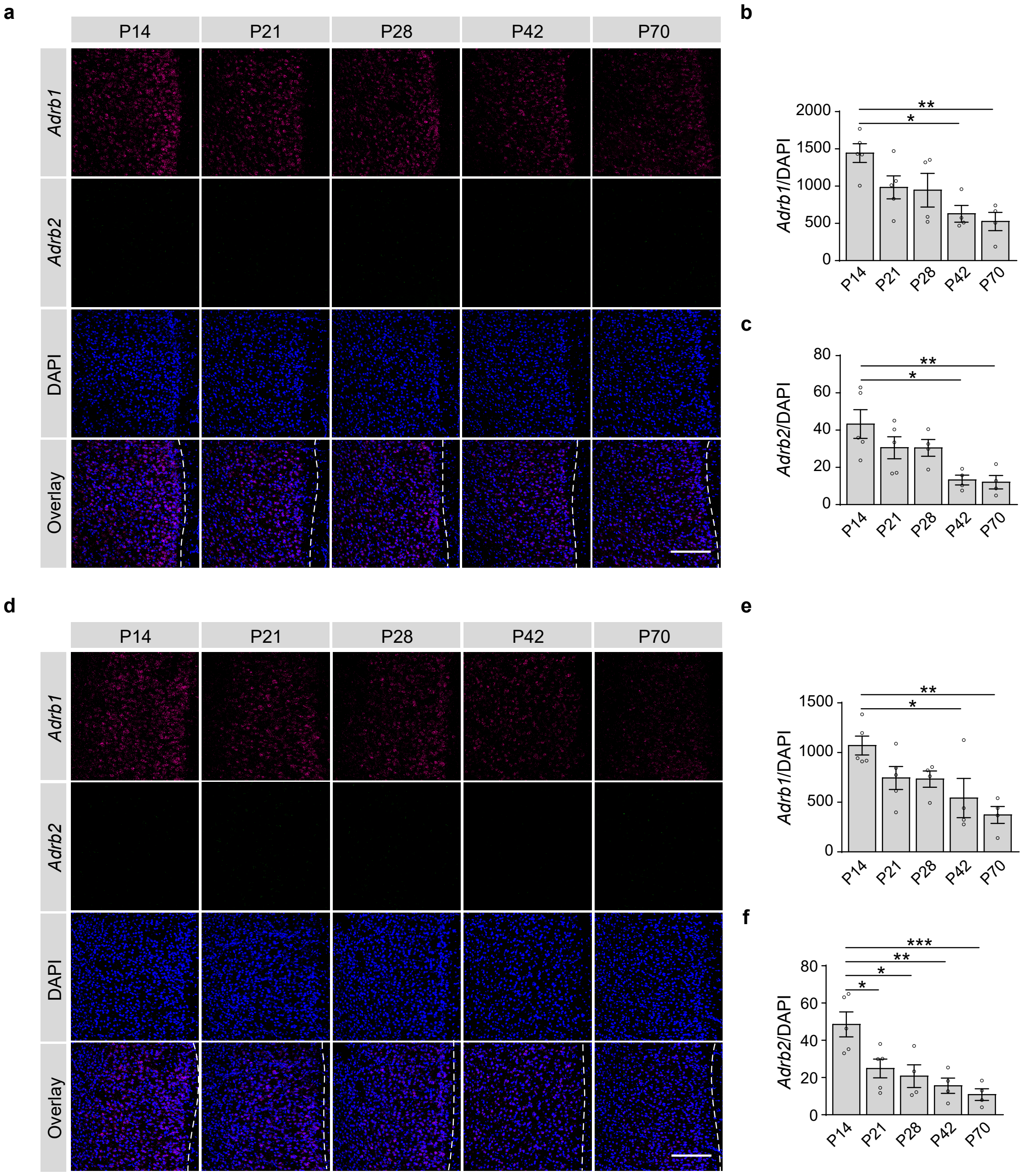

Supplementary Fig. 12 *Adrb1* and *Adrb2* mRNA expression in the PrL and IL of juvenile and adult mice. Fluorescence in situ hybridization of *Adrb1* and *Adrb2* mRNA was performed with the brain slices containing the PrL and IL of C57 mice (P14, P21, P28, P42, P70). **a,d** Representative images of *Adrb1* and *Adrb2* mRNA expression in the PrL and IL. **b,c,e,f** Statistical graph for of *Adrb1* and *Adrb2* mRNA expression levels in the PrL and IL [P14: $n = 5$; P21: $n = 5$; P28: $n = 4$; P42: $n = 4$; P70: $n = 4$. PrL: *Adrb1*: $F_{(4,17)} = 5.74$, $p = 0.0041$; *Adrb2*: $F_{(4,17)} = 5.57$, $p = 0.0047$. IL: *Adrb1*: $F_{(4,17)} = 4.82$, $p = 0.0088$; *Adrb2*: $F_{(4,17)} = 7.81$, $p < 0.001$]. Scale bar: 200 μm. $^{*}p < 0.05$, $^{**}p < 0.01$, $^{***}p < 0.001$ vs indicated group.

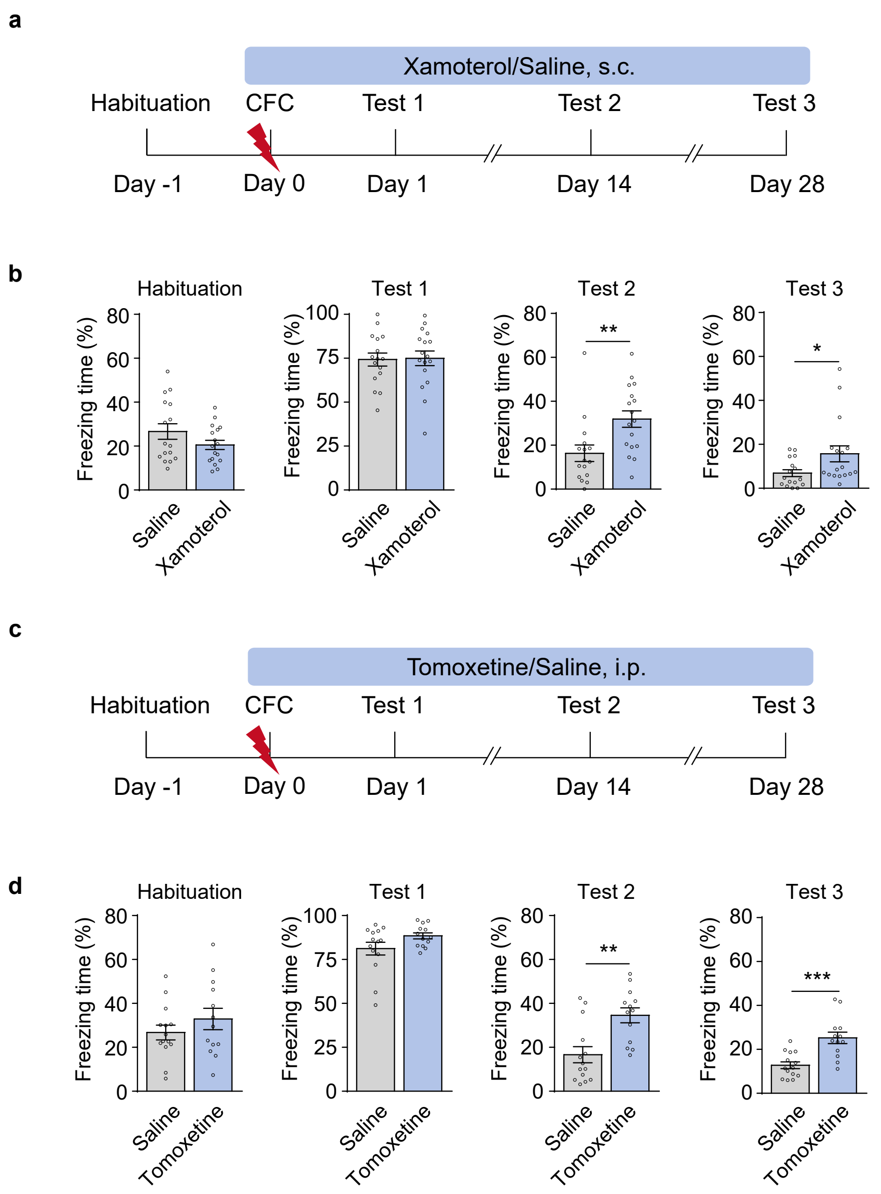

Supplementary Fig. 13 Activation of adrenergic signaling before and after CFC improves remote memory storage in juvenile mice. a,c Experimental scheme. Xamoterol (3 mg/kg, s.c.) or Tomoxetine (3 mg/kg, i.p.) was continuously injected for 28 days in the juvenile mice. Memory retention tests were performed 1, 14, and 28 days after CFC. **b,d** Statistical graphs for freezing levels [**b**: Saline: $n = 16$; Xamoterol: $n = 17$; Test 2: $U = 54, p = 0.0025$; Test 3: $U = 76, p = 0.030$; **d**: Saline: $n = 14$; Tomoxetine: $n = 13$; Test2: $U = 30$, $p = 0.0023$; Test3: $t_{(25)} = 4.20$, $p < 0.001$]. $^{*}p < 0.05$, $^{**}p < 0.01$, $^{***}p < 0.001$ vs indicated group.
