# Supplementary material for: Noradrenergic signaling mediates cortical early tagging and storage of remote memory
Source: Nat Commun. 2022 Dec 9;13:7623. doi: 10.1038/s41467-022-35342-x (PMC9734098; doi:10.1038/s41467-022-35342-x)
Supplement: Supplementary file 3 — Description of Additional Supplementary Files [file 41467_2022_35342_MOESM3_ESM.pdf]

Description of Additional Supplementary Files

Supplementary Data 1: Mouse lines and viral vectors

Supplementary Data 2: Statistics

Supplementary Software 1: These codes are MATLAB scripts which can run based on MATLAB environment. The codes can be used to analyze the NE release in the mPFC, DG, BLA, and NAc during footshock and optical stimulation.
