# Supplementary material for: Noradrenergic signaling mediates cortical early tagging and storage of remote memory
Source: Nat Commun. 2022 Dec 9;13:7623. doi: 10.1038/s41467-022-35342-x (PMC9734098; doi:10.1038/s41467-022-35342-x)
Supplement: Supplementary file 6 — Supplementary Software 1 [file 41467_2022_35342_MOESM6_ESM.zip › Readme.pdf]

使用说明

推荐使用 Matlab 2017b 运行该软件。

1.运行 Matlab，使用 1 处的按钮将路径设置在分析程序根目录。

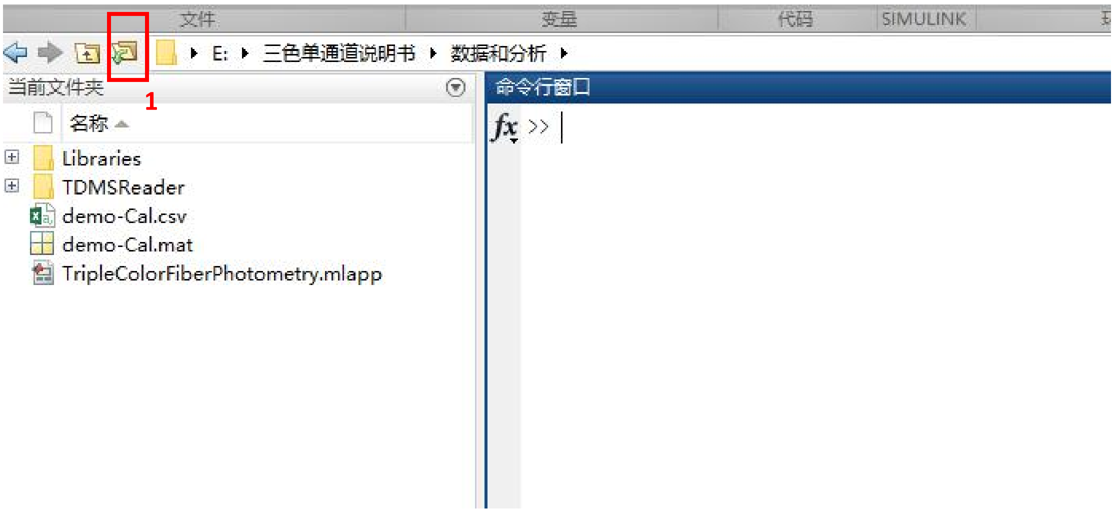

2.双击左侧目录中 TripleColorFiberPhotometry.mlapp 文件，或使用鼠标右击选择运行打开分析软件。

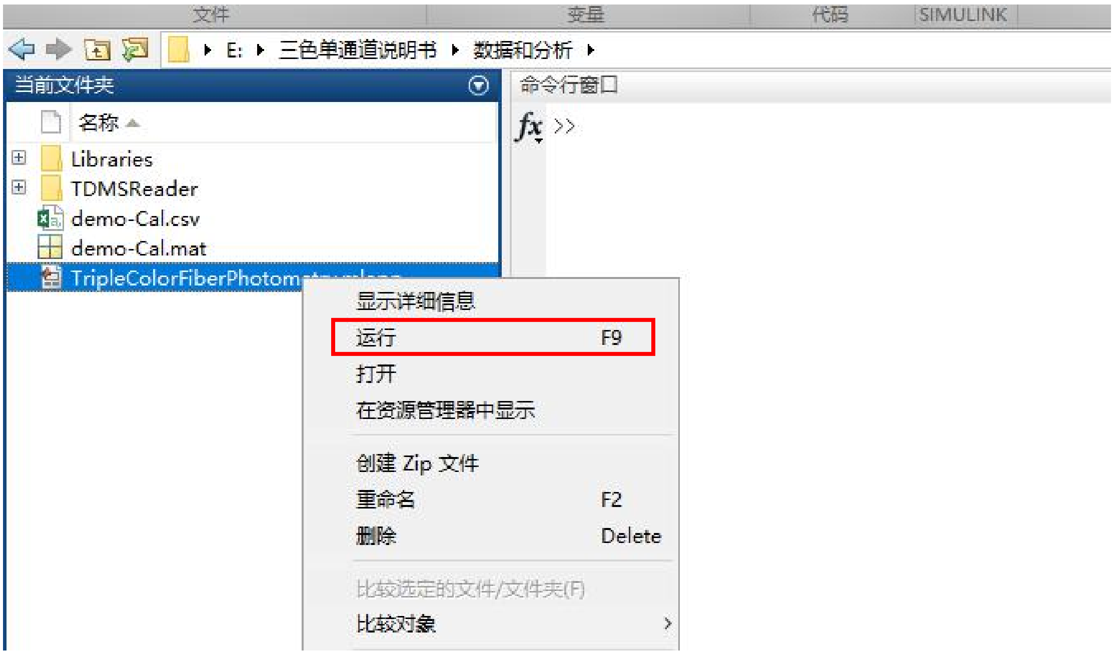

3、弹出如图所示的界面，界面包含两个模块，分别如图所示。

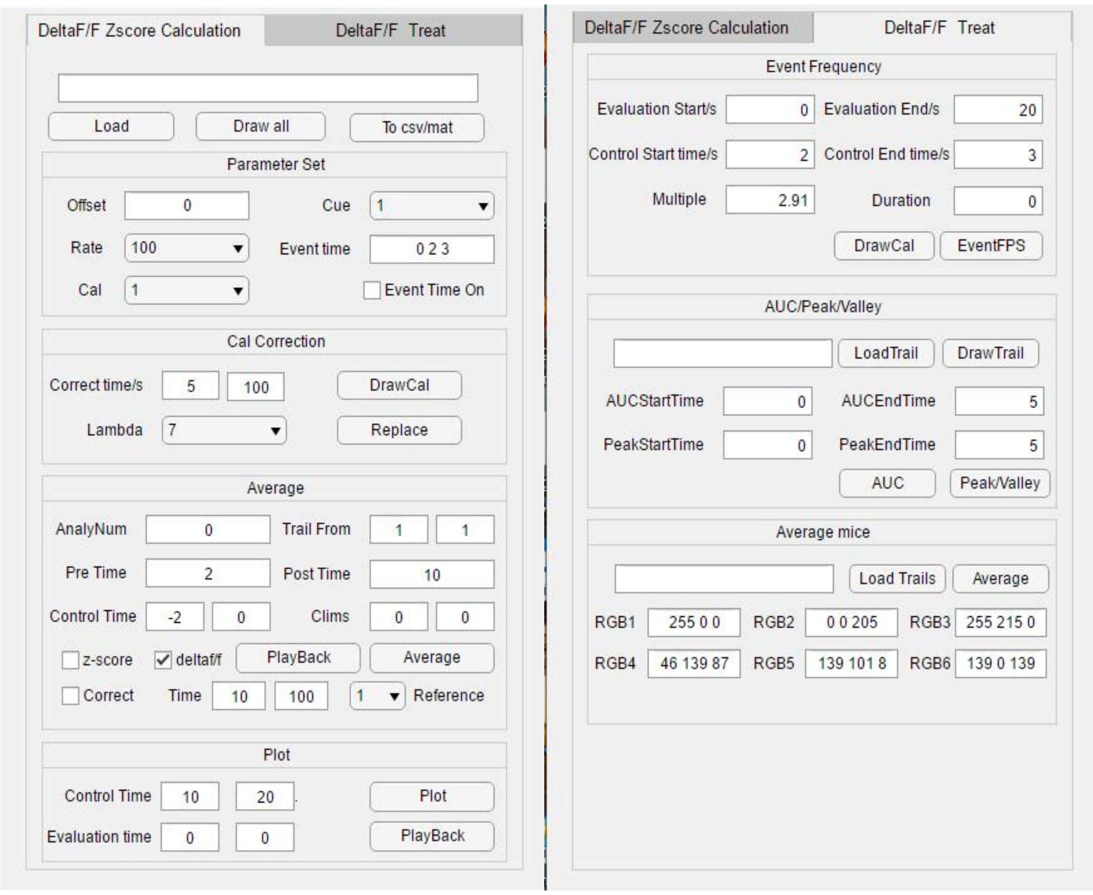

4、单击 Load 按钮选择数据采集的数据，如图所示。弹出数据读取完成提示框表示数据读取成功。

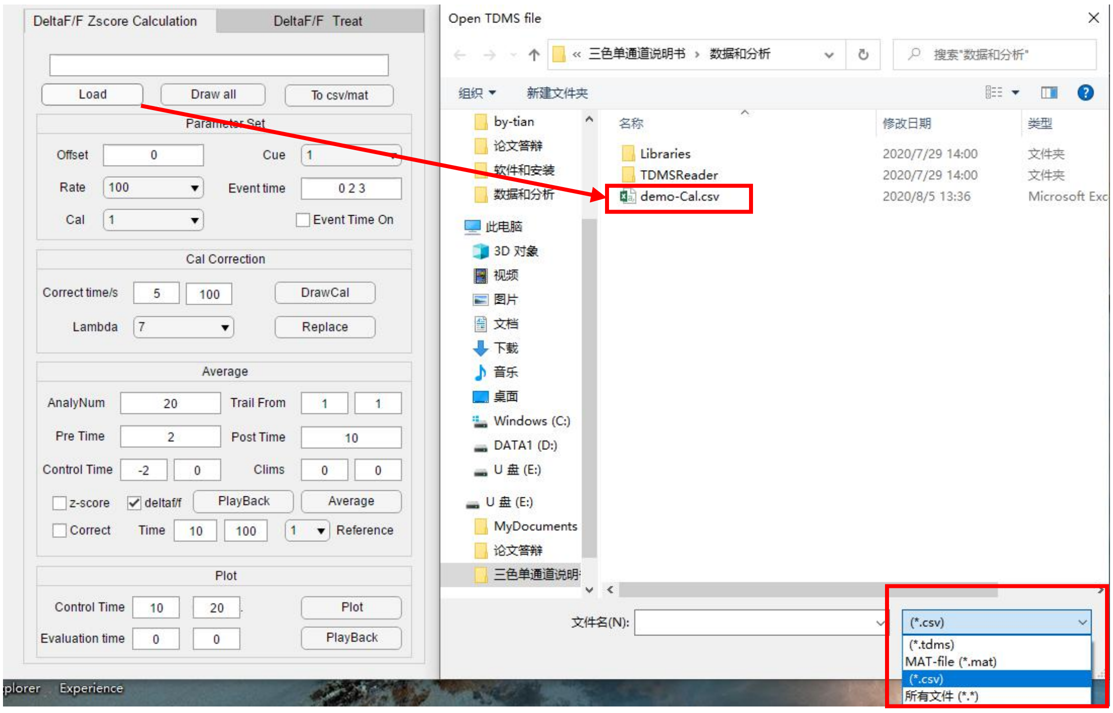

5、单击 Draw all 按钮，绘制采集的信号全部数据曲线图。

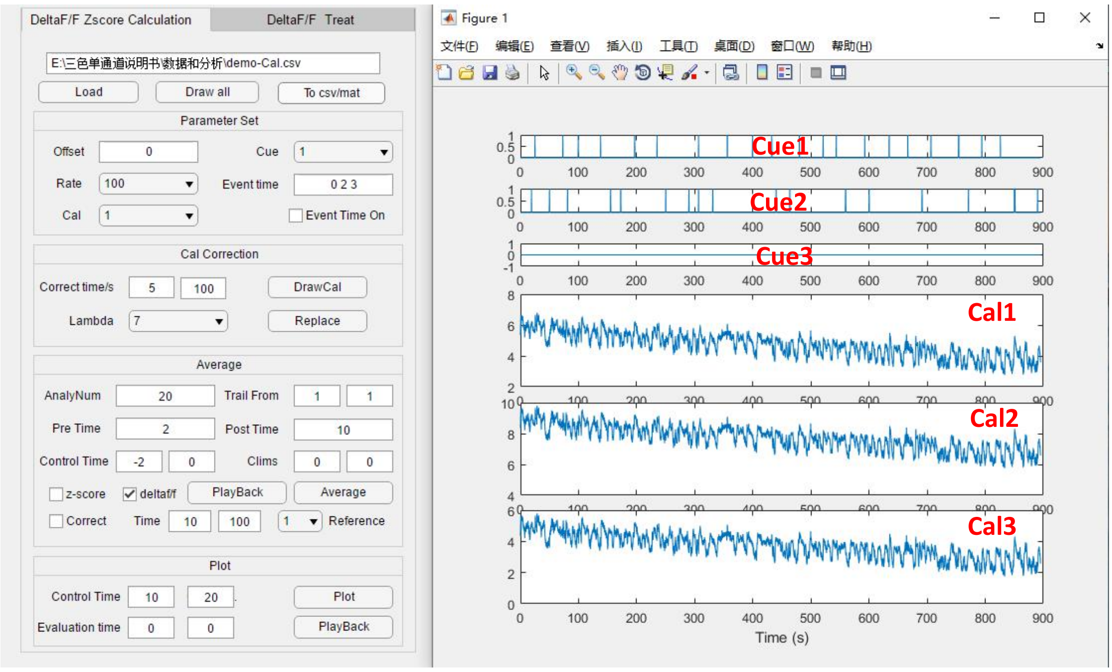

单击 To csv/mat 按钮可把读取数据中的 tdms 文件（采集卡采集的格式是 tdms 格式）转换成 csv 和 mat 格式（也可直接读取 tdms 文件进行分析）

6、参数设置

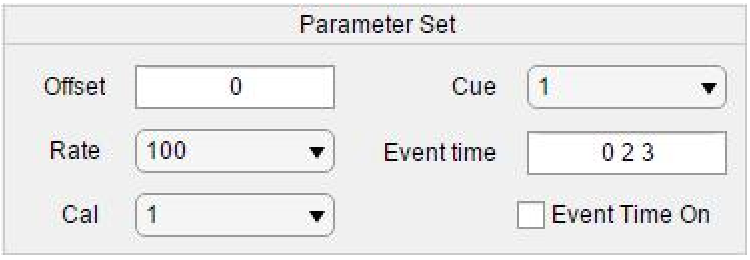

（1）Offset：将设备所有参数调整完成后，将光纤末端放置于黑暗环境中，此时采集软件对应通道的数值是系统的偏置信号，这里称为对应通道的钙信号的 Offset 值（输入参数数目应与钙信号通道数目一致，中间用空格或者逗号间隔）；

（2）Rate：钙信号的采样频率，应与采集软件设定的频率一致，一般默认是的 100Hz；

（3）Cal：用于选择当前分析钙信号通道（1 通道或者 2 通道），通道序号应对应采集软件中的 ROI 编号。

（4）Cue: 非人工打标，选择当前分析参考的事件通道，此时不要选中参数 Event Time/Cue；

（5）Event Time：人工打标，填写标记位置的时间，如 20.1 30.5 单位为秒，多个时间之间用空格隔开；

（6）Event Time On 选择框：选中时，可以使用 EventTime 输入的参数时间点作为打标时刻，否则即为 Cue 方式打标

7、钙信号矫正

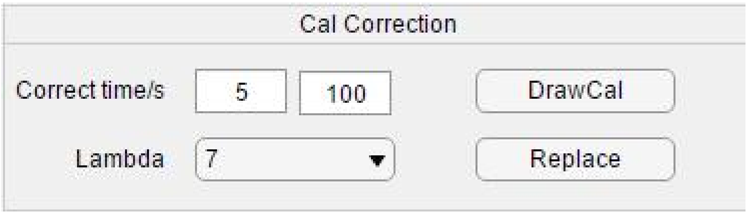

使用算法对原始钙信号进行矫正，恢复原始数据在记录过程中因为 GCamp 或类似荧光蛋白漂白造成的信号趋势下降的问题，矫正算法参照附录文献。

（1） Correct time/s：矫正开始与结束时间。

根据矫正算法，该区间选择记录的动物的钙信号数据为宜，即动物插上光纤后到拔下光纤前的数据，否则混乱的环境光信号会影响矫正结果。

（2） DrawCal：查看选择通道的钙信号数据以及矫正时间区间。

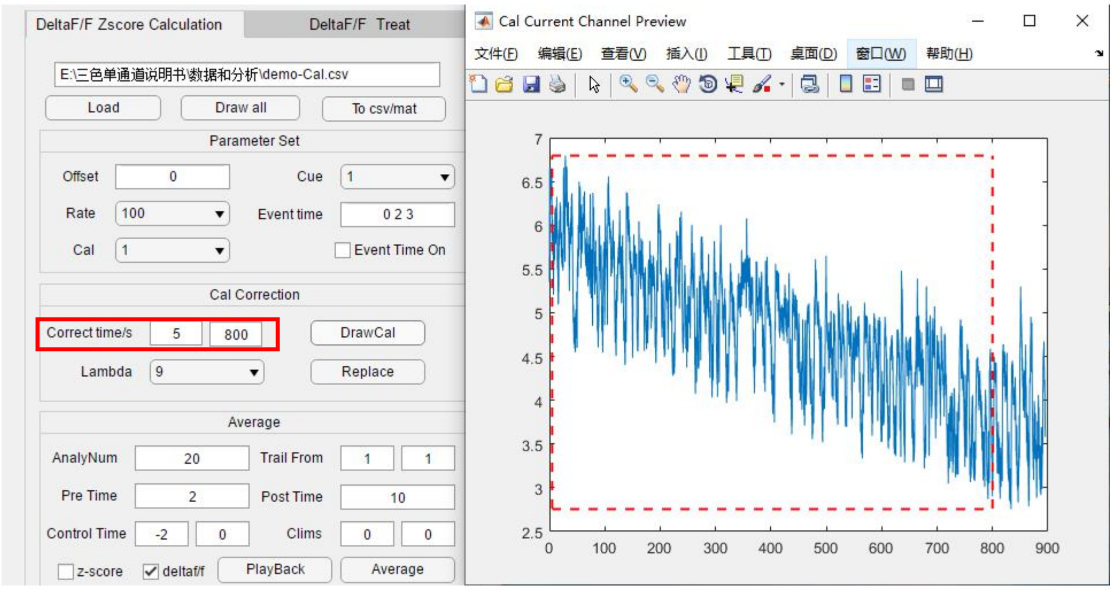

（3） Lambda：表示矫正系数，选择参数后会自动弹出矫正效果图，矫正结果正常为保留原始信号变化的同时下降趋势矫正，不同的数据需要使用不同的系数 Lambda 进行矫正，该系数 Lambda 的设置通常在 7-9 之间，可以通过 1 处的功能按钮放大数据查看，根据矫正结果选择最合适的矫正参数 Lambda。

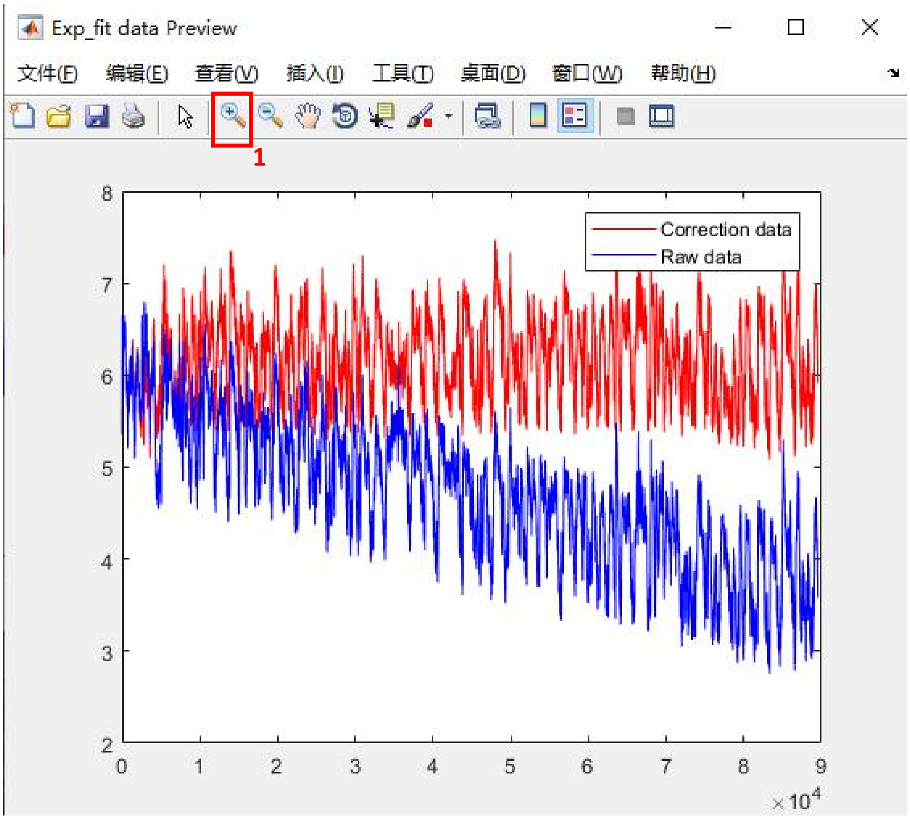

合适的矫正系数 Lambda 参数，矫正后数据保留原始数据变化

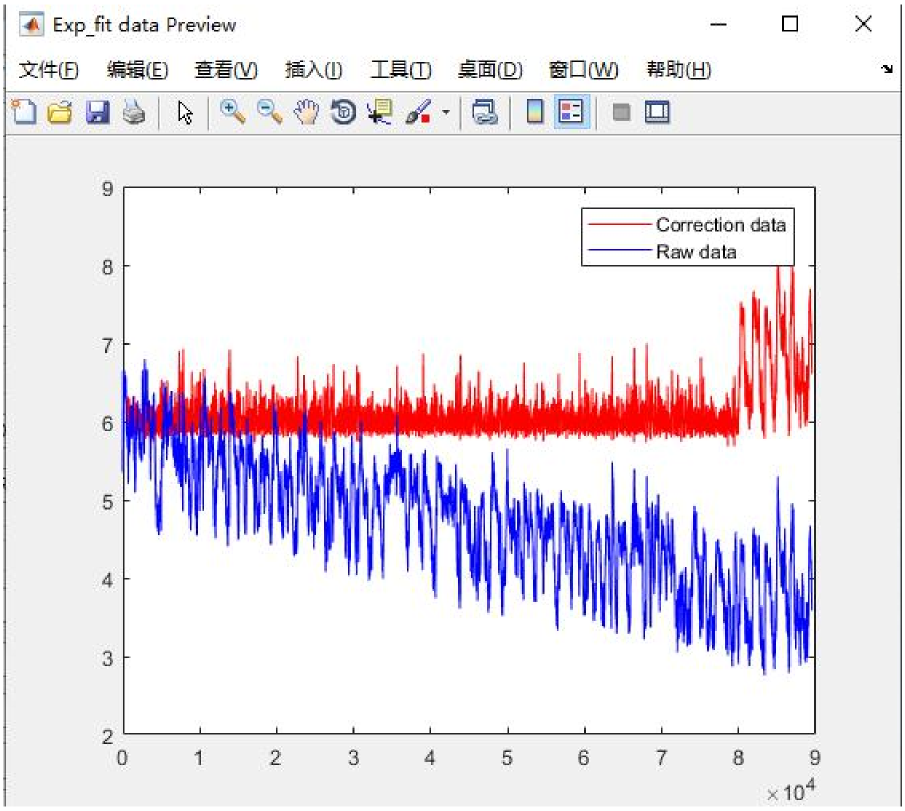

不合适的 Lambda 参数

（4） Replace：使用 Replace 按钮使用矫正后的数据替换原始数据进行后续数据分析，此时点击读取数据部分的 Draw all 按钮即可查看修正后的钙信号数据，同时将在数据同路径下生成一个 XXX_Cal_Correction 文件，文件保存了矫正后的数据以及矫正参数信息。

注意：a.程序只会矫正(1)中设置的时间窗内的数据，时间窗外的数据将在保留原始变化的前提下和矫正后数据做拼接。

b.替换数据操作一旦替换无法取消，此时如果要切换回原始数据分析，可以在读取数据时使用 Load Cal 按钮重新读取原始钙信号数据文件即可，也可以读取生成的 Cal_Correction 文件读取校正后的数据进行分析。

8、Average 模块

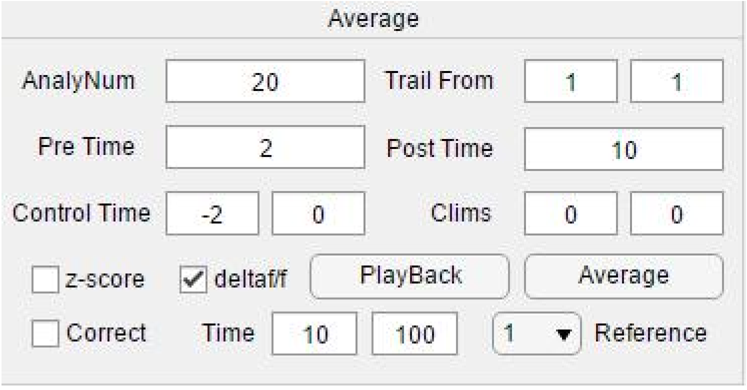

进行多个 Trial 的平均值分析

该分析方法用来展示在多次重复性实验中神经元钙信号活动的重复性结果，该方法需要记录动物的行为事件的时间戳，以时间戳将信号（图中 Stim 时间点）分为多个不同的 Trial，然后再将每一段钙信号数据单独做 Deltaf/F 或 Z-score 的分析计算。

该分析方法一般用于评价动物在某一特定重复性试验下的钙信号结果分析，分析算法如下：

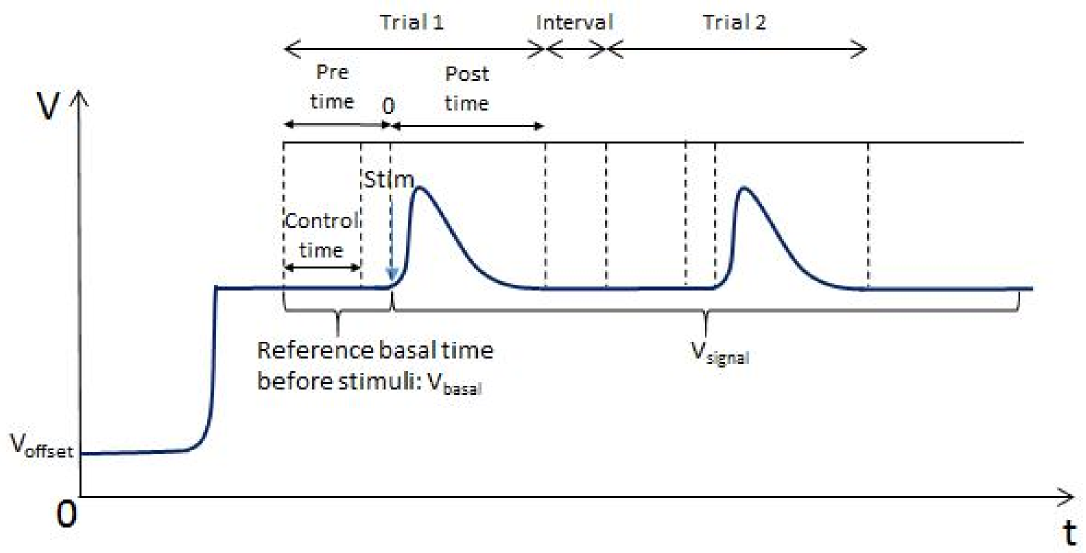

$$F_0 = \overline{V_{basal}} \qquad \sigma_F = \mathrm{STD}(V_{basal})$$

$$\frac{\Delta F}{F_0} = \frac{V_{signal} - F_0}{F_0 - V_{offset}} \qquad \text{Z-score} = \frac{V_{signal} - F_0}{\sigma_F}$$

$\overline{V_{basal}}$: average value of V_{basel} in reference time;
σ_F: standard deviation of V_{basel} in reference time;

（1）AnalyNum：要分析的 Trial 数目；

（2）Trail From：设定需要分析的 Trial 区间；

（3）Pre time：设定分析窗口相对于刺激前的时间；

（4）Post time：设定分析窗口相对于刺激后的时间；

（5）Control time：设定计算 F0 的参考时间窗口（输入数据为区间），以每一次行为事件的时间戳时间为 0 时间；

（6）Clims：设定绘制 heatmap 的颜色对应数值区间，设置为 0 时程序将自动计算最合适的值适配结果；

（7）z-score/deltaF/F：设定分析评价指标类型；

（8）correct：选择结合参考通道进行矫正 ，同时在 time 框输入矫正参考时间窗（选择比较平稳上小鼠之后一段基线时间范围）；

（9）Reference 选择框：使用 correct 矫正时选择的参考通道，如果参考通道和 Cal 通道相同，则不矫正；

（10）average 按钮：输出分析结果；

（11）PlayBack 按钮：可选择对 average 分析保存过的数据进行图形复现。

如图所示：

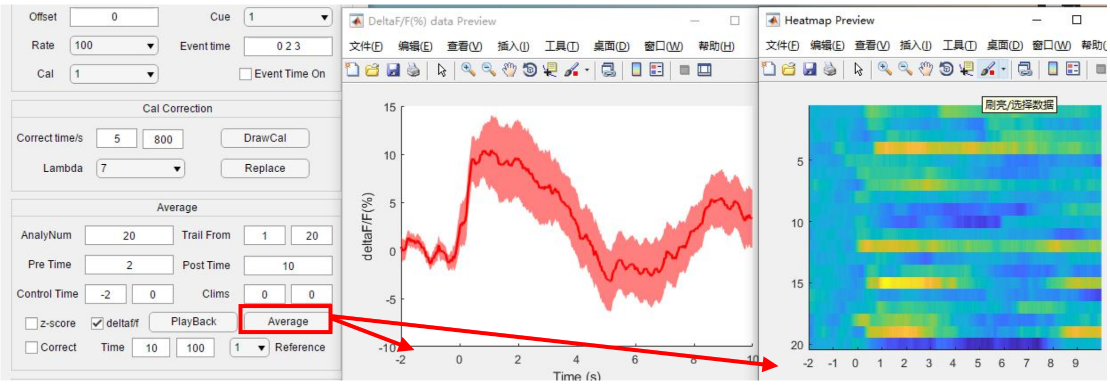

右图的 Heatmap 图显示了在多次重复的 Trial 实验中每一次结果统计出的结果数据。

中间的曲线图显示了所有 Trial 结果的平均值结果，阴影部分为每个 Trial 数据间的 SEM（标准差）运算，即阴影部分越小，说明每个 Trial 的数据大小一致性较高，反之亦然。

9、Plot 模块

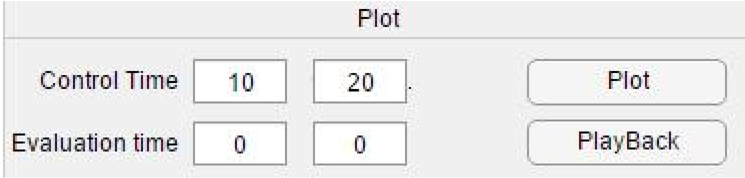

该方法用以对指定时间窗的数据做 Deltaf/F 或 Z-score 的换算。

该方法一般用于反应实验动物在行为或刺激后的神经元长时间内的活动变化，用来评价一些不可重复的非急性变化过程对神经元活动的影响。

该分析方法一般用于评价动物在某一非急性干预试验下的钙信号结果分析，一般用来评价神经元钙信号水平在长时间神经调控下的信号，分析算法如下：

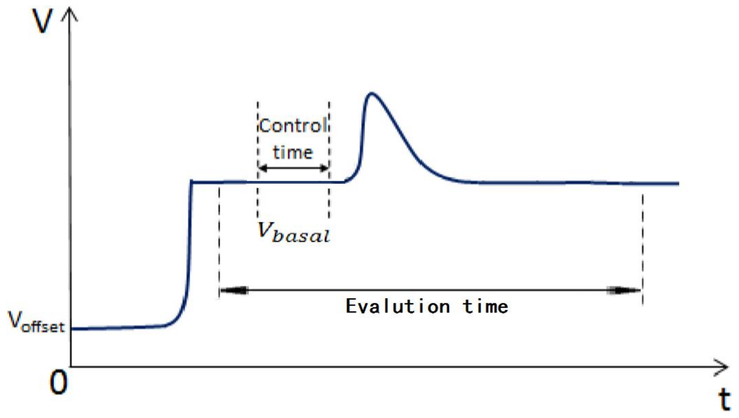

$$F_0 = \overline{V_{basal}}$$

$$\frac{\Delta F}{F_0} = \frac{V_{signal} - F_0}{F_0 - V_{offset}}$$

$$\sigma_F = \mathrm{STD}(V_{basal})$$

$$\text{Z-score} = \frac{V_{signal} - F_0}{\sigma_F}$$

（1）Control time：设定计算 F0 的参考时间窗口，以原始数据的 0 时间为 0 时间；

（2）Evaluation time：设定待分析的数据时间窗，以原始数据的 0 时间为 0 时间，设置为 0 时将选取所有数据作为分析时间区间；

（3）Plot 按钮：完成一个区间内数据的分析，如图所示；

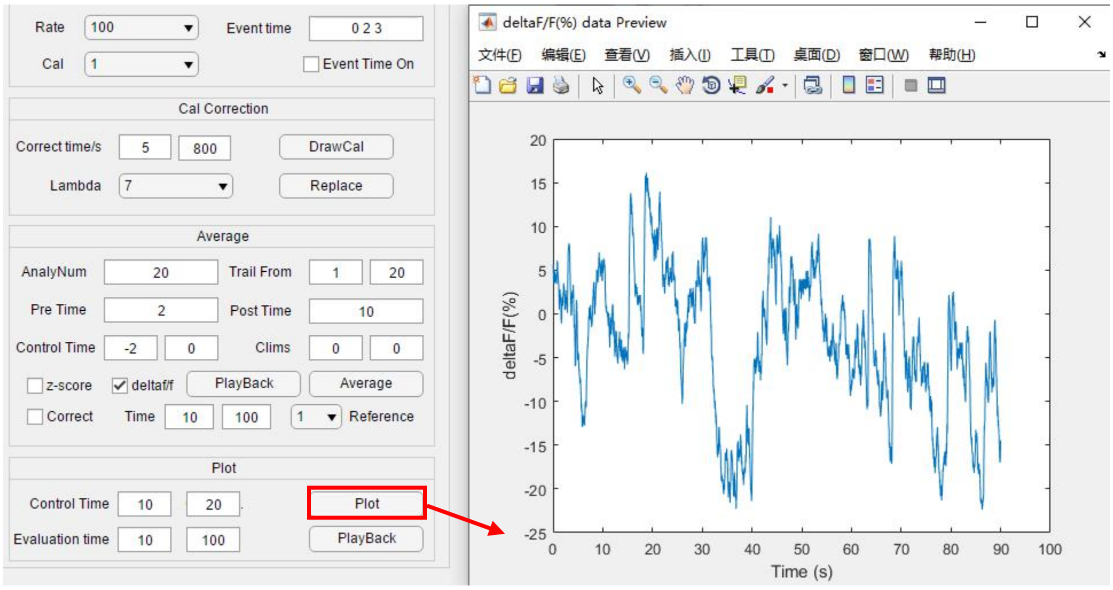

（4）PlayBack 按钮：可选择对 Plot 分析保存过的数据进行图形复现。

10、 Deltaf/f Treat 模块分析

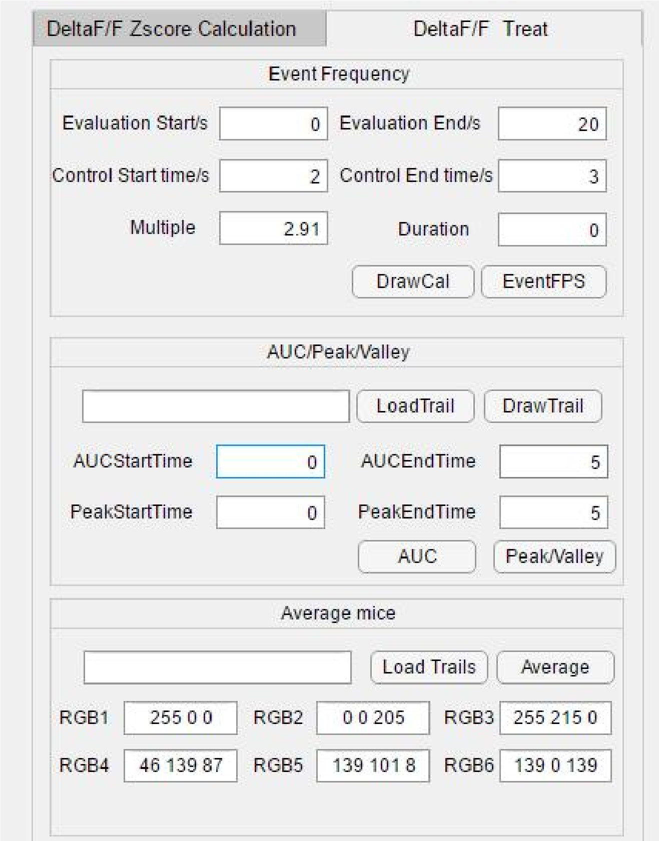

11、Event Frequency 模块

该方法通过计算原始数据和基线值（F0）的比值差异，当原始信号的值大于一定倍数的基线值且持续了一定时间时，则认为钙信号出现了一次因为行为（事件）引起的钙信号，该分析方法用来统计发生行为钙信号的次数和时间。

分析算法如下：

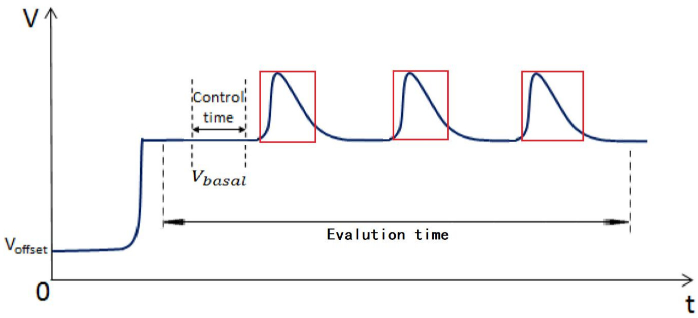

$$F_0 = \overline{V_{basal}}$$

$$V_{signal} > \text{Mutiple} \times F_0$$

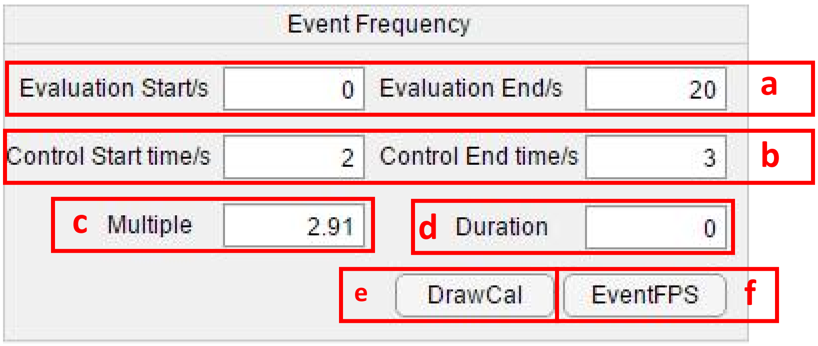

a) 设置需要分析的数据时间窗，单位：秒，以原始数据的 0 时间为 0 时间，设置为 0 时将选取所有数据作为分析时间区间。

b) 设定计算 F0 的参考时间窗，单位：秒，以原始数据的 0 时间为 0 时间。

c) 设定分析的比值系数（当钙信号大于 F0 的系数倍时，认为行为造成了一次钙信号）。

d) 设定认定发生事件的最小持续时间（小于该时间则认为虽然满足倍数要求，但是因为持续时间较短所以不足以认定为一次钙信号发放）。

e) 查看选择通道的钙信号数据、参考时间窗（Control Time）以及分析窗（Evaluation Time）的时间区间。

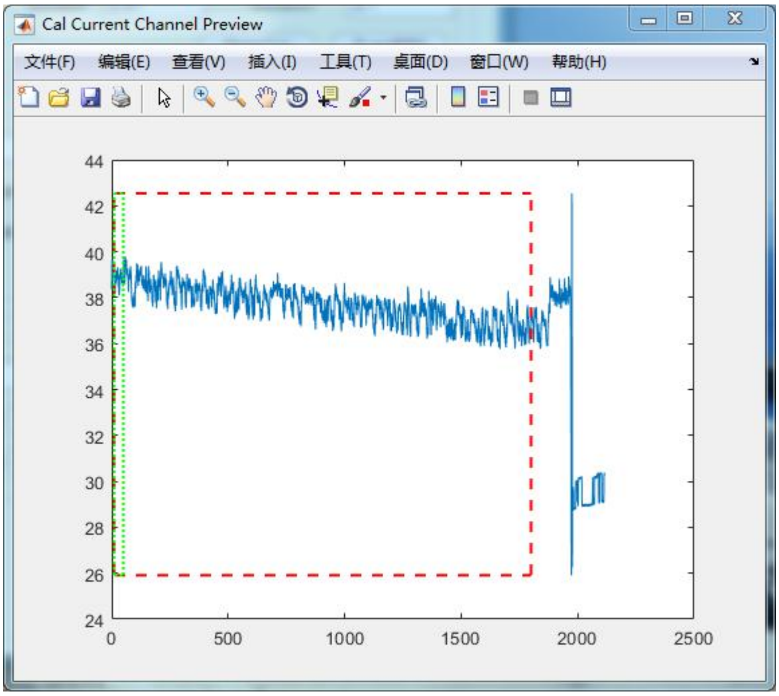

绿框和红框分别显示了参考窗和分析窗的时间区间

f) 计算行为（事件）发生时间和频率。

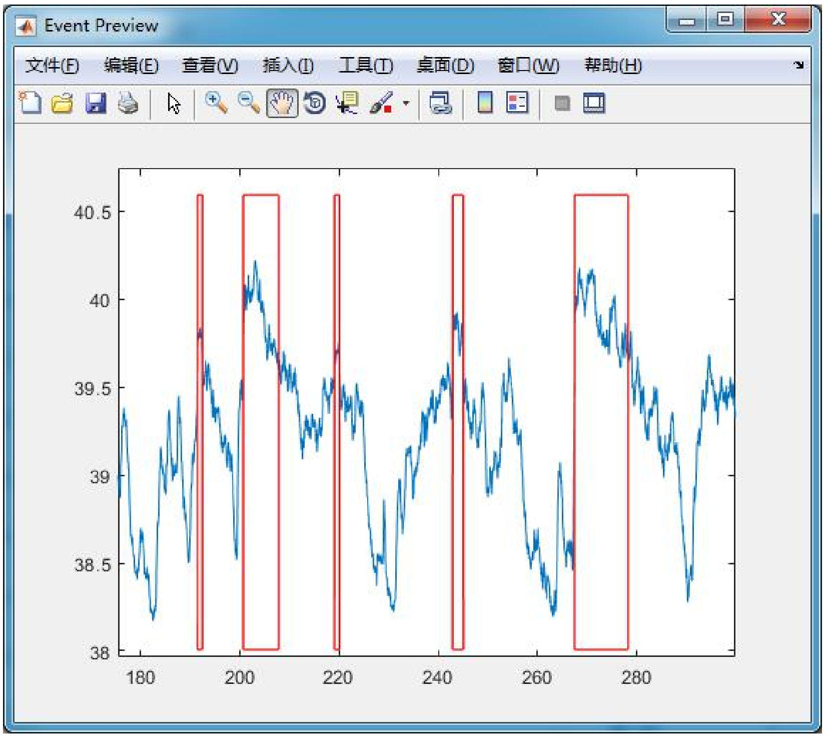

红框表示该时间数据满足事件发生要求

结果数据和设置参数可以自定义文件名称进行保存，如果不保存，数据也会被自动保存在原始数据同目录下的 XXX_Event_FPS 文件中（后续覆盖）。

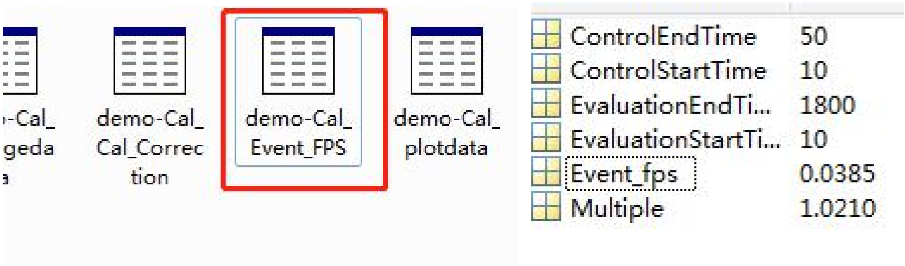

12、AUC/Peak/Valley 模块

计算多个 Trial 分析结果（averagedata）每一个 Trial 的线下面积以及平均值结果的最大和最小值以及最大和最小值出现的时间。

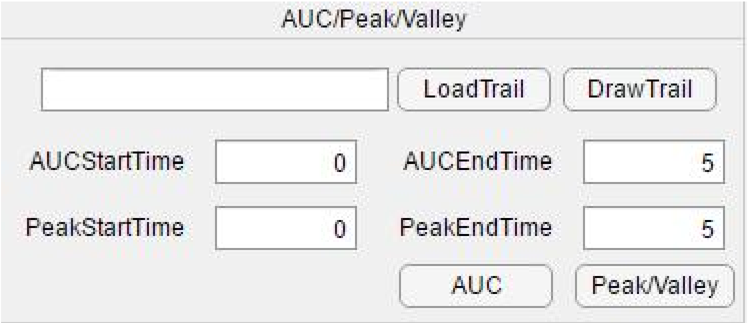

（1）该模块基于计算过后的 Deltaf/f 数据进行分析(即 DeltaF/F Zscore Calculation 模块，单击 average 生成的数据)，首先单击 Load trial 按钮选择，trail 数据。如图所示。弹出数据完成提示框表示数据读取成功。

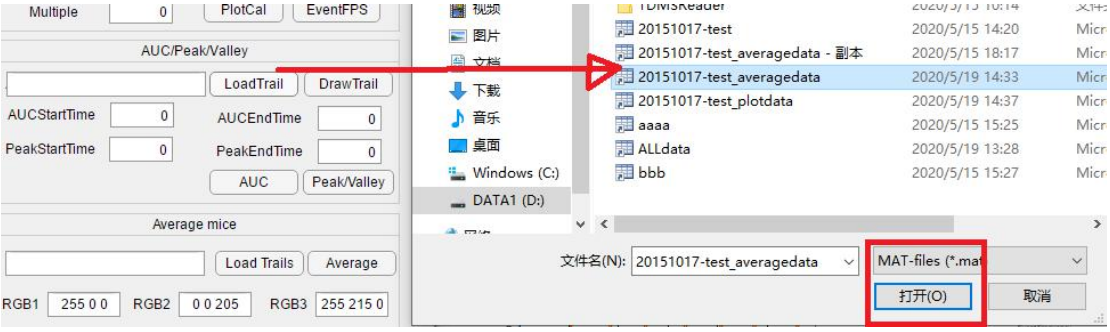

（2）单击 Draw Trail 可预览选择 trial 文件的数据图，如图所示。

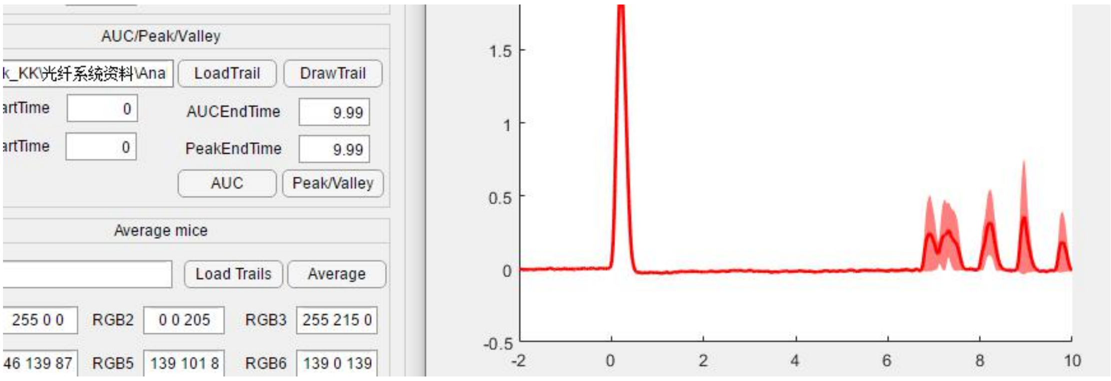

（3）AUC Starttime 以及 AUCendtime 设定想要计算的 AUC 的开始时间与结束时间，单击 AUC 按钮计算 AUC（线下面积）值并保存。如图所示。

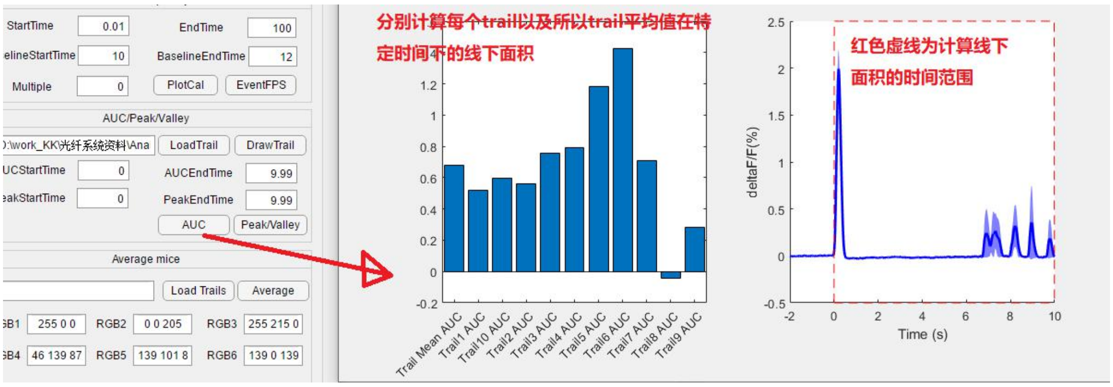

数据保存为：20171018_averagedata_AUC.mat （读取数据的文件名_AUC.mat）。

（4）Peak/Valley 设定想要分析的起始时间分别填入 peak Start time 以及 peak End time 当中。单击 Peak/Valley 按钮进行计算。得到的结果保存格式如图。

20171018_averagedata_Peak.mat 原始名称加上后缀_Peak.mat。

12、Average mice 模块

将多只动物的 Trial 的平均值结果进行统计汇总。

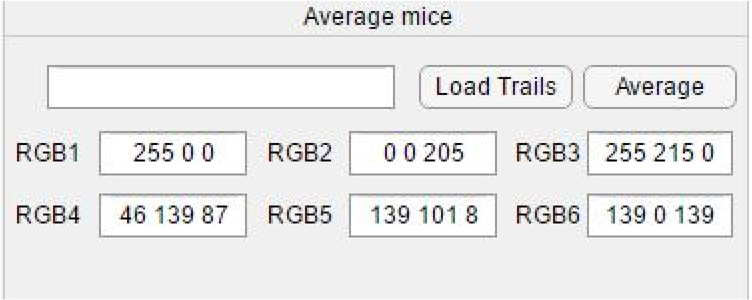

（1）单击 Load Trials 按钮，选择多只老鼠的数据，如图所示。

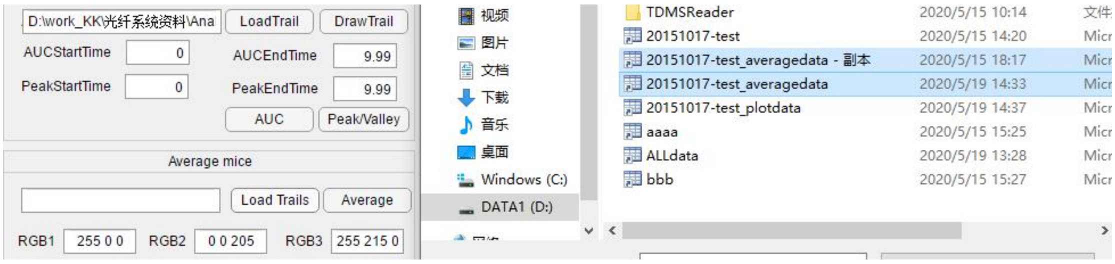

（2）单击 averagea 按钮，如图，其中 RGB1 RGB2 RGB3 RGB4 RGB5 RGB6 表示对多只老鼠或者多个 trail 合并在一个坐标轴画图时，颜色设定，如，选择 3 个数据时，使用 RGB1-3 的颜色；当选择 7 个数据时，使用 RGB1-6 以及 RGB1 的数据循环添加颜色。RGB 颜色可自定义，RGB 格式必须是 0-255 范围的三个数据。

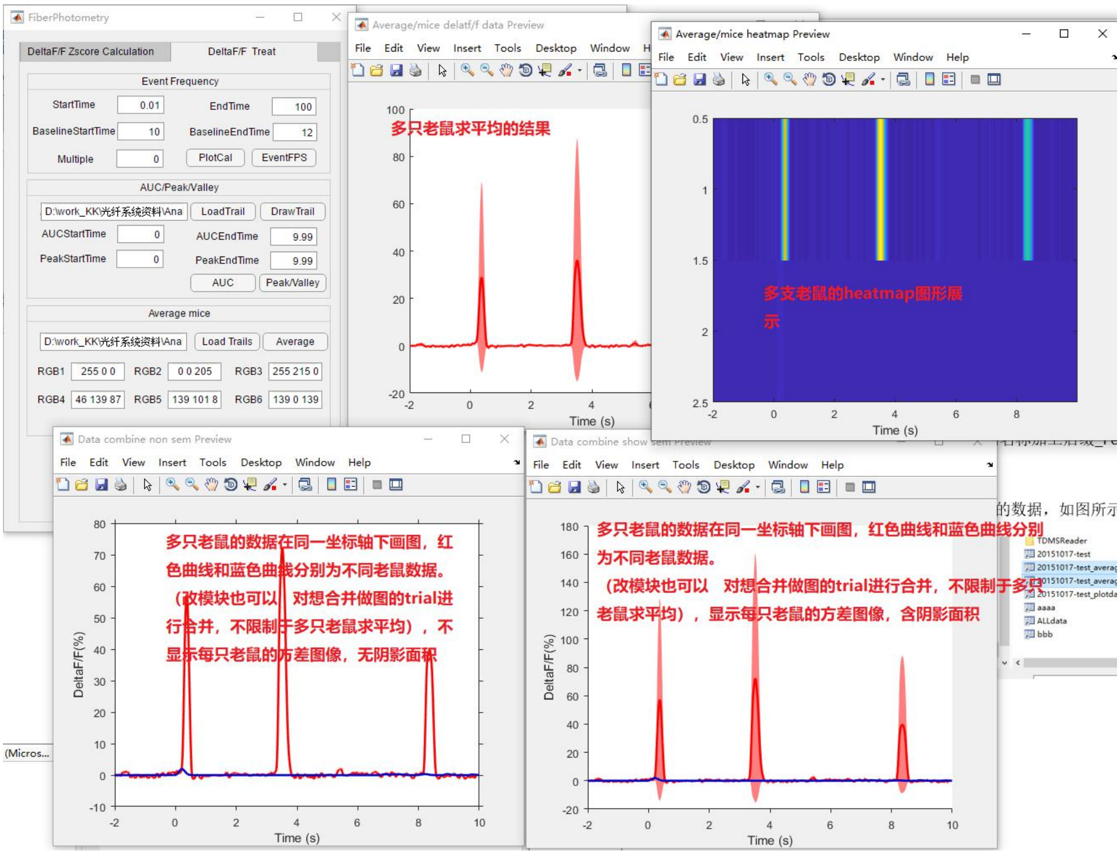
FiberPhotometry
DeltaF/F Zscore Calculation
DeltaF/F Treat
Event Frequency
StartTime
EndTime
BaselineStartTime
BaselineEndTime
Multiple
PlotCal
EventFPS
AUC/Peak/Valley
LoadTrail
DrawTrail
AUCStartTime
AUCEndTime
PeakStartTime
PeakEndTime
AUC
Peak/Valley
Average mice
Load Trails
Average
Average/mice delatf/f data Preview
多只老鼠求平均的结果
Time (s)
Average/mice heatmap Preview
多支老鼠的heatmap图形展示
Data combine non sem Preview
多只老鼠的数据在同一坐标轴下画图，红色曲线和蓝色曲线分别为不同老鼠数据。（改模块也可以 对想合并做图的trial进行合并，不限制于多只老鼠求平均），不显示每只老鼠的方差图像，无阴影面积
DeltaF/F(%)
Data combine show sem Preview
多只老鼠的数据在同一坐标轴下画图，红色曲线和蓝色曲线分别为不同老鼠数据。
（改模块也可以 对想合并做图的trial进行合并，不限制于多只老鼠求平均），显示每只老鼠的方差图像，含阴影面积
