# Supplementary material for: Noradrenergic signaling mediates cortical early tagging and storage of remote memory
Source: Nat Commun. 2022 Dec 9;13:7623. doi: 10.1038/s41467-022-35342-x (PMC9734098; doi:10.1038/s41467-022-35342-x)
Supplement: Supplementary file 7 — Reporting Summary [file 41467_2022_35342_MOESM7_ESM.pdf]

nature portfolio

Corresponding author(s): Xing Liu, Lan Ma

Last updated by author(s): Nov 11, 2022

Reporting Summary

Nature Portfolio wishes to improve the reproducibility of the work that we publish. This form provides structure for consistency and transparency in reporting. For further information on Nature Portfolio policies, see our Editorial Policies and the Editorial Policy Checklist.

Statistics

For all statistical analyses, confirm that the following items are present in the figure legend, table legend, main text, or Methods section.

n/a	Confirmed	
☐	☒	The exact sample size (*n*) for each experimental group/condition, given as a discrete number and unit of measurement
☐	☒	A statement on whether measurements were taken from distinct samples or whether the same sample was measured repeatedly
☐	☒	The statistical test(s) used AND whether they are one- or two-sided<br>*Only common tests should be described solely by name; describe more complex techniques in the Methods section.*
☐	☒	A description of all covariates tested
☐	☒	A description of any assumptions or corrections, such as tests of normality and adjustment for multiple comparisons
☐	☒	A full description of the statistical parameters including central tendency (e.g. means) or other basic estimates (e.g. regression coefficient) AND variation (e.g. standard deviation) or associated estimates of uncertainty (e.g. confidence intervals)
☐	☒	For null hypothesis testing, the test statistic (e.g. *F*, *t*, *r*) with confidence intervals, effect sizes, degrees of freedom and *P* value noted<br>*Give P values as exact values whenever suitable.*
☒	☐	For Bayesian analysis, information on the choice of priors and Markov chain Monte Carlo settings
☒	☐	For hierarchical and complex designs, identification of the appropriate level for tests and full reporting of outcomes
☒	☐	Estimates of effect sizes (e.g. Cohen's *d*, Pearson's *r*), indicating how they were calculated

Our web collection on statistics for biologists contains articles on many of the points above.

Software and code

Policy information about availability of computer code

Data collection	Behavioral experiments were recorded using MED-PC IV (Med Associates, Inc.).<br>Photometry recording for NE release was performed using ThinkerTech Fiber photometry Acquisition System (ThinkerTech Nanjing Biotech).<br>Microscopy data was collected with Nikon A1 or Olympus DP80 Application Suite software (NIS-AR V5.02 or CellSens V1.13).
Data analysis	Fear conditioning: Video Freeze® software provided by MED Associates.<br>Cell counting and IOD calculations: Image-Pro Plus 6.0 software.<br>Statistics: SPSS_v20 and MATLAB R2020a.<br>Matlab code used for NE release analysis is available in the Supplementary information-MATLAB code.

For manuscripts utilizing custom algorithms or software that are central to the research but not yet described in published literature, software must be made available to editors and reviewers. We strongly encourage code deposition in a community repository (e.g. GitHub). See the Nature Portfolio guidelines for submitting code & software for further information.

Data

Policy information about availability of data

All manuscripts must include a data availability statement. This statement should provide the following information, where applicable:

- Accession codes, unique identifiers, or web links for publicly available datasets
- A description of any restrictions on data availability
- For clinical datasets or third party data, please ensure that the statement adheres to our policy

All source data generated in this study are provided in the Supplementray information-Source data.

Human research participants

Policy information about studies involving human research participants and Sex and Gender in Research.

Reporting on sex and gender	This study did not involve human research participants.
Population characteristics	This study did not involve human research participants.
Recruitment	This study did not involve human research participants.
Ethics oversight	This study did not involve human research participants.

Note that full information on the approval of the study protocol must also be provided in the manuscript.

Field-specific reporting

Please select the one below that is the best fit for your research. If you are not sure, read the appropriate sections before making your selection.

- [x] Life sciences
- [ ] Behavioural & social sciences
- [ ] Ecological, evolutionary & environmental sciences

For a reference copy of the document with all sections, see nature.com/documents/nr-reporting-summary-flat.pdf

Life sciences study design

All studies must disclose on these points even when the disclosure is negative.

Sample size	No statistical methods were used to predetermine sample size. Our sample sizes were estimated based on our previous experience (e.g. Yiming Zhou et al., 2019, Nat Neurosci) and are similar to those generally employed in the field. Detailed sample size was described in the Supplementary information-Statistics.
Data exclusions	In the experiments with virus injection, animals with missed virus injection site were excluded.
Replication	All the experiments were reliably reproduced using at least two parallel experiments.
Randomization	For animal experiments, age-matched male mice were randomly assigned to experimental or control groups before surgery and experiments.
Blinding	Blinding was done for behavioral tests and cell counting during data collection and analysis, groups were masked until the end of experiment.

Reporting for specific materials, systems and methods

We require information from authors about some types of materials, experimental systems and methods used in many studies. Here, indicate whether each material, system or method listed is relevant to your study. If you are not sure if a list item applies to your research, read the appropriate section before selecting a response.

Materials & experimental systems

n/a	Involved in the study	
☐	☒	Antibodies
☒	☐	Eukaryotic cell lines
☒	☐	Palaeontology and archaeology
☐	☒	Animals and other organisms
☒	☐	Clinical data
☒	☐	Dual use research of concern

Methods

n/a	Involved in the study	
☒	☐	ChIP-seq
☒	☐	Flow cytometry
☒	☐	MRI-based neuroimaging

Antibodies

Antibodies used

The following antibodies were used for Immunofluorescence (IF) and immunohistochemistry (IHC).
Rabbit anti-c-Fos antibody (Santa Cruz, sc-52, 1:1000, Lot: F1715).
Mouse anti-TH antibody (Millipore, MAB318, 1:1000, Lot: 3202369).
Alexa Fluor 488 goat anti-rabbit IgG (Jackson ImmunoResearch, 111-545-144, 1:1000).
Cy3 goat anti-rabbit IgG (Jackson ImmunoResearch, 111-165-144, 1:1000).
Alexa Fluor 488 goat anti-mouse IgG (Jackson ImmunoResearch, 115-545-466, 1:1000).
Cy3 goat anti-mouse IgG (Jackson ImmunoResearch, 115-165-166, 1:1000).
Biotin-conjugated anti-mouse IgG (Jackson ImmunoResearch, 715-065-150, 1:200).

Validation

Primary antibodies:
anti-c-Fos: PMID: 26023136.
anti-TH: PMID: 25664911.

Animals and other research organisms

Policy information about studies involving animals; ARRIVE guidelines recommended for reporting animal research, and Sex and Gender in Research

Laboratory animals

Male 6-8 week-old Wild-type C57BL/6 mice were obtained from Shanghai Laboratory Animal Center, CAS.
TH-Cre mice (#008601) were purchased from The Jackson Laboratory (CA, USA).
AI14 reporter mice (#007914) were purchased from The Jackson Laboratory (CA, USA).
Fos2A-iCreER mice (#030323) were purchased from The Jackson Laboratory (CA, USA).
ArcCreER mice (#021881) were purchased from The Jackson Laboratory (CA, USA).
c-fos-tTA mice (#018306) were purchased from The Jackson Laboratory (CA, USA).
Drd1fiox/flox mice (#025700) were purchased from The Jackson Laboratory (CA, USA).
Dbh-Cre mice (#036778) were purchased from MMRRC (MO, USA).
Adrb1fiox/flox mice were developed by our lab.
Adrb2fiox/flox mice were developed by our lab.
H2B-GFP mice were gifts from M He (Institutes of Brain Science, Fudan University, China).
Adult mice (P70) and juvenile mice (P20) were used for behavioral experiments in our study.

Wild animals

This study did not involve wild animals.

Reporting on sex

Only male mice were used for behavioral experiments.

Field-collected samples

This study did not involve field collected samples.

Ethics oversight

All experimental procedures were approved by the Animal Care and Use Committee of the Shanghai Medical College of Fudan University.

Note that full information on the approval of the study protocol must also be provided in the manuscript.
